# Sex-Specific Fifteen-Year Alcohol Consumption Trajectories and Their Association with Cardiovascular Events and Mortality: The Framingham Heart Study

**DOI:** 10.3390/nu18050849

**Published:** 2026-03-05

**Authors:** Yuanming Leng, Huitong Ding, Yi Li, Xue Liu, Mengyao Wang, Yumeng Cao, Chenglin Lyu, Daniel Levy, Jiantao Ma, Chunyu Liu

**Affiliations:** 1 Department of Biostatistics, Boston University School of Public Health, Boston, MA 02118, USA; lengym@bu.edu (Y.L.); liyij@bu.edu (Y.L.); xliu168@bu.edu (X.L.); mengyaow@bu.edu (M.W.); yumeng88@bu.edu (Y.C.); 2 Department of Anatomy and Neurobiology, Boston University Chobanian & Avedisian School of Medicine, Boston, MA 02118, USA; dinghfut@bu.edu (H.D.); clynk@bu.edu (C.L.); 3 Framingham Heart Study, Boston University Chobanian & Avedisian School of Medicine, Boston, MA 02118, USA; 4 Population Sciences Branch, National Heart, Lung, and Blood Institute, National Institutes of Health, Bethesda, MD 20892, USA; levyd@nih.gov; 5 Framingham Heart Study, National Heart, Lung, and Blood Institute (NHLBI), Framingham, MA 01702, USA; 6 Nutrition Epidemiology and Data Science, Friedman School of Nutrition Science and Policy, Tufts University, Boston, MA 02111, USA

**Keywords:** alcohol consumption, cardiovascular disease, coronary heart disease, mortality, trajectory analysis, survival analysis

## Abstract

**Background**: Alcohol use patterns influence health outcomes. This study examined sex-specific drinking trajectories and their associations with all-cause mortality and coronary heart disease (CHD) in the US-based Framingham Heart Study. **Method**: Among 6570 participants (mean age: 55 ± 13; 55% women) followed for 15 years, a growth mixture model identified four sex-specific alcohol consumption trajectories. Cox models examined associations of alcohol trajectories with CHD and mortality over 10 years of follow-up, adjusting for covariates. **Results**: This study identified four distinct, sex-specific alcohol consumption trajectories: the Moderate-Decreasing group (1179 women, 0–14 g/day; 1534 men, 0–28 g/day) showed a declining moderate intake, The Low-to-None group included light or non-drinkers (992 women, 826 men), the Inverse-U group (606 women, 199 men) showed variable intake over time, while the High-Decreasing group (858 women, 376 men) had high initial consumption (women > 14 and men > 28 g/day) that declined over time. Compared with the Moderate-Decreasing group, women in other groups had higher CHD risks (HRs 1.58–1.61) and greater mortality risk in the Low-to-None (HR 1.25) and Inverse-U (HR 1.28) groups. Men in Low-to-None had higher mortality (HR 1.17) and CHD (HR 1.60), while High-Decreasing showed the highest mortality (HR 1.27). Low-to-moderate drinking was associated with lower mortality and CHD risks; however, these findings do not confirm the protective effects of alcohol use. **Discussion**: Our findings suggest that sustained low to moderate drinking was associated with lower risks of mortality and CHD in both women and men, compared to high-level or fluctuating patterns. Although these associations may not confirm causality, our findings emphasize the importance of investigating long-term drinking patterns in public health. Nevertheless, we caution against promoting moderate alcohol use as a strategy to reduce mortality risk or prevent CHD.

## 1. Introduction

Alcohol consumption is a major contributor to the global disease burden [1]. Extensive research has shown that the relationship of alcohol consumption with mortality and cardiovascular disease (CVD) is complex and multifaceted [2,3,4]. Evidence suggests that alcohol-related cardiovascular effects are non-linear rather than uniformly protective or harmful [5,6]. Studies, including the Global Burden of Disease study (1990 and 2016) [1], reported a J-shaped association in which light-to-moderate drinking is associated with the lowest risk, particularly among middle-aged and older participants [7,8,9]. In addition, women are more biologically susceptible to alcohol-related harm due to differences in body composition, metabolism, and hormone profiles [10]. Consequently, similar levels of intake may result in higher organ vulnerability compared with men, likely contributing to sex-specific and non-linear cardiovascular associations [11]. Previous studies show divergent associations between alcohol consumption and hypertension in women, where occasional drinking is associated with a lower risk, whereas frequent drinking increases risk compared to abstinence [12]. Despite growing evidence, sex-specific differences in the long-term effects of alcohol on mortality and CVD risk remain poorly understood [13,14].

Concerns about confounding and selection bias, particularly among participants who abstain due to pre-existing health conditions, challenge the notion that moderate drinking is protective [15]. Most existing studies are cross-sectional or assess alcohol consumption at a single point in time, which may not capture the dynamic nature of drinking habits that often change with aging, health status, and health-related stressors [16,17]. Increase in alcohol consumption, particularly transitions to heavy drinking, is associated with a higher risk of mortality and CVD [7]. Conversely, reductions in alcohol consumption may reflect either intentional lifestyle improvements or underlying declining health conditions prompting cessation [18]. Together, these changes highlight the importance of longitudinal studies to understand how sustained and evolving alcohol consumption is associated with long-term cardiovascular risk.

Recent large cohort studies have examined life-course trajectories of alcohol consumption and their associations with mortality and cardiometabolic outcomes [19,20,21]. However, sex-specific trajectory patterns across generations and their associations with mortality and CVD remain incompletely characterized. To address these gaps, our study examines sex-specific patterns of long-term alcohol consumption and their associations with mortality and CVD outcomes, particularly coronary heart disease (CHD, the major type of CVD). We hypothesize that distinct alcohol consumption trajectories exist over time in men and women, and that these trajectory groups are associated with varying all-cause mortality and CHD risks. Using data from the Framingham Heart Study (FHS), a long-term community-based cohort originating in Massachusetts, USA, we applied sex-specific growth mixture modelto identify alcohol consumption trajectories (Aim 1) and conducted sex-specific multivariable-adjusted regression analyses to evaluate their associations with all-cause mortality and CHD (Aim 2). These analyses aim to improve our understanding of how long-term drinking patterns influence disease risk in men and women.

## 2. Method

**Figure 1:**
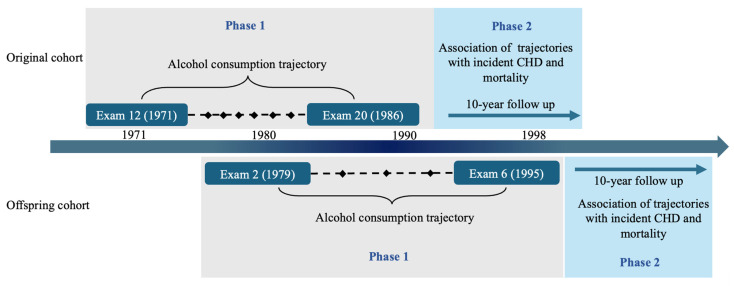
Study timeline and data collection milestones. The FHS is a long-term, community-based, multi-generational cohort study in Framingham, Massachusetts, USA. To investigate cardiovascular risk factors, the Original cohort (*n* = 5209) was recruited in 1948, followed by the recruitment of their offspring and the spouses of the offspring into the Offspring cohort (*n* = 5124) in 1971. The present analysis included all available participants from the Original cohort and Offspring cohort who attended exam 12 (Original cohort) and exam 2 (Offspring cohort). The year on the arrow reflects the longitudinal nature of the Original and Offspring cohorts in the FHS. In Phase 1, alcohol consumption trajectories were constructed using longitudinal alcohol consumption data collected over 15 years between 1971 and 1986 for the Original cohort and between 1979 and 1995 for the Offspring cohort. In Phase 2, association analyses were conducted to examine the relationship between trajectory groups and incident CHD and mortality, with a 10-year follow-up period.

We analyzed longitudinal alcohol consumption data and outcome variables in two phases: Phase 1 identified drinking trajectories over 15 years, and Phase 2 assessed their associations with mortality and CHD risk (Figure 1).

### 2.1. Study Participants

The FHS is a long-term, community-based, multi-generational cohort study in Framingham, Massachusetts, USA. The participants in FHS underwent in-person exams at regular intervals, including medical history assessments, physical exams, laboratory tests, and lifestyle questionnaires [22,23]. To investigate cardiovascular risk factors, the Original cohort (*n* = 5209) was recruited in 1948, followed by the recruitment of their offspring and the spouses of the offspring into the Offspring cohort (*n* = 5124) in 1971. The participants in the Original cohort underwent up to 32 exams every 2 to 4 years, and those in the Offspring cohort have completed up to 10 exams, with exams conducted every 4 years on average(with an 8-year gap between the first two). This study was approved by the Institutional Review Board at Boston University Medical Center (Protocol H-41461). All procedures complied with ethical guidelines and regulations (Supplemental Document). All participants provided written informed consent.

The present analysis initially included all available participants from the Original cohort and Offspring cohort who attended exam 12 (Original cohort, mean age 64 ± 8, range: 50–91 at baseline of Phase 1) and exam 2 (Offspring cohort, mean age 44 ± 10, range: 17–72 at baseline of Phase 1). Including both cohorts is crucial for studying potential generational differences in alcohol consumption and CVD outcomes. These differences may reflect the impact of changes over time in socioeconomic conditions, access to medical care, and lifestyle factors, including alcohol consumption, CVD prevention, and treatment practices.

**Figure 2:**
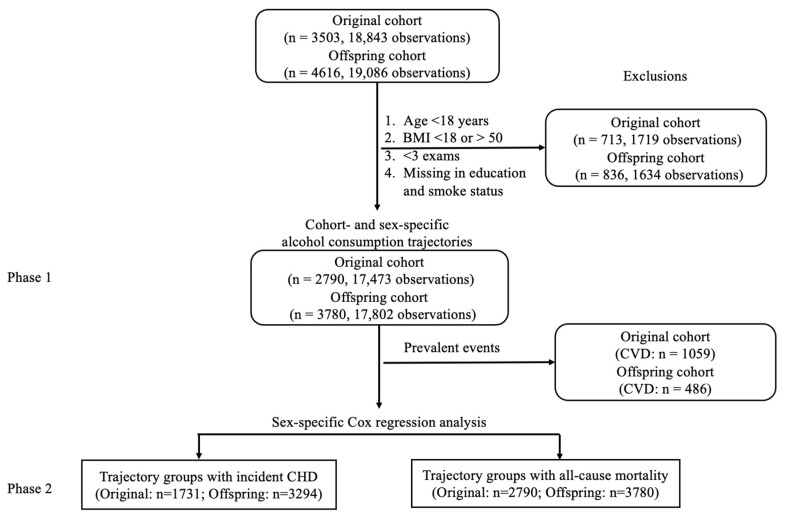
Study design. The present analysis initially included all available participants from the Original cohort and Offspring cohort who attended exam 12 (Original cohort) and exam 2 (Offspring cohort). Several exclusion criteria were applied to select participants (*n* = 2790 from the Original cohort and *n* = 3780 from the Offspring cohort) for the alcohol consumption trajectory analysis. In Phase 1, cohort- and sex-specific trajectory analysis was conducted to identify alcohol consumption trajectory groups. In Phase 2, sex-specific Cox proportional hazards regression was performed to evaluate if alcohol trajectory groups were associated with total mortality and CHD. Prevalent events refer to participants who developed CVD or CHD before the baseline of Phase 2 analysis.

For the Original cohort, we included participants from eight exams collected over 15 years (Figure 1): Exam 12 (1971–74, baseline), exams 13, 14, 15, 17, 18, 19, and Exam 20 (1986–90). Data prior to Exam 12 were excluded due to inconsistent or incomplete data collection on the types and frequency of alcoholic beverage consumption. Exam 16 data were also excluded because alcohol consumption data were not available. For the Offspring cohort, data from five exams over 16 years were included (Figure 1): Exam 2 (1979–1983, baseline) and exams 3, 4, 5, and 6 (1995–1998). Exam 1 was excluded because the data on the measurement of alcoholic beverage consumption were inconsistent with those in the later exams. In total, 3503 participants (18,843 observations) from the Original cohort and 4616 participants (19,086 observations) from the Offspring cohort were included in subsequent analyses (Figure 2).

Several additional exclusion criteria (Figure 2) were applied to the data before trajectory analysis in Phase 1. We excluded participants under 18 years of age at baseline (*n* = 6) because they undergo significant physiological development. We also excluded participants with a BMI below 18 kg/m^2^ (62 participants, 0.9% of total sample size) or above 50 kg/m^2^ (8 participants, 0.1% of total sample size), as these extremes may reflect underlying health conditions or atypical physiology that could bias trajectory patterns or outcome associations, consistent with prior recommendations [24,25,26]. In addition, to ensure longitudinal consistency, we excluded participants who attended fewer than three examinations, had missing alcohol consumption data, or lacked covariate information at two or more consecutive examination visits. After applying the exclusion criteria, 713 participants from the Original cohort and 836 from the Offspring cohort were removed, leaving 6570 participants (2790 from the Original cohort and 3780 from the Offspring cohort) for the Phase 1 analysis to identify alcohol consumption trajectory patterns (Figure 1 and Figure 2). At the baseline of Phase 2, participants with prevalent CVD outcomes (i.e., any existing CVD cases identified prior to or at the baseline of Phase 2) were excluded from further analysis for incident CHD. A total of 1545 prevalent CVD cases, including 1076 prevalent CHD cases, were excluded (Figure 1 and Figure 2).

In the final analytic sample, overall missingness was approximately 2%. Alcohol consumption and covariates demonstrated strong correlations between adjacent examinations (e.g., alcohol consumption: r = 0.7; BMI: r = 0.8, both *p* < 0.001). To maximize sample size, missing values were imputed by averaging measurements from the two nearest examinations, assuming the missing data were due to sporadic absences (i.e., missing at random) (Figure 2). Because trajectory classification was based on multiple repeated measures, a single imputed value was unlikely to substantially influence group assignment.

### 2.2. Alcohol Consumption Measurement

Lifestyle information, including alcohol consumption, was collected at most exams through a standalone, technician-administered questionnaire [27]. Participants were asked about their average weekly consumption of beer, wine, or liquor (in a standard portion size) over the past year. A standard drink was 12 ounces of beer, 4 to 5 ounces of wine, or 1.5 ounces of liquor, each containing approximately 14 grams of ethanol [28] (Supplemental Methods). Given the right-skewed distribution of alcohol consumption and the high proportion of participants without alcohol intake, a cohort- and sex-specific Box–Cox transformation was applied to the alcohol consumption data to improve normality and support more stable regression estimates [29] (Supplemental Figure S1).

### 2.3. Definition of All-Cause Mortality, CHD, and CVD

All-cause mortality was defined as death from any cause. CVD outcomes (including CHD) and death events were adjudicated using standardized FHS procedures. A panel of three physicians reviewed medical records and death certificates to ensure accurate and consistent diagnoses [30]. CHD was defined as recognized and unrecognized myocardial infarction, angina pectoris, coronary insufficiency, and CHD death, based on clinical evaluation, electrocardiographic findings, biomarkers, and relevant documentation, as previously described [31,32]. CVD comprised CHD, stroke, heart failure (HF), and death from any cardiovascular condition [33]. For the analysis of all-cause mortality, follow-up time was calculated from the Phase 2 baseline to the earliest of the following events: date of death, date of last contact, or 10 years post-baseline, whichever occurred first. For incident CHD, follow-up time was calculated from the Phase 2 baseline to the earliest of the following: the first occurrence of a CHD event, date of last contact, death, or 10 years post-baseline.

### 2.4. Covariates

In Phase 1, covariates included baseline education and smoking status assessed at each exam in the trajectory analysis of alcohol consumption. Education status was categorized into four levels: no high school, high school, some college, and college graduate. Current smoking status at each exam was recorded as a binary variable (Yes or No), indicating whether the participant was smoking regularly in the past year before an exam.

In Phase 2, covariates include education level, smoking status, systolic blood pressure (SBP), hypertension treatment, diabetes, and BMI at the baseline of Phase 2 [34]. Except for education level, all covariates used in the Phase 2 analysis were measured at the baseline of Phase 2. We used the same education-level variable in both phases, as our study sample consisted primarily of middle-aged participants. Systolic blood pressure (SBP) was measured twice by physicians, and the average of the two readings was used in our analysis [30]. Diabetes was defined as a fasting glucose level of 126 mg/dL or greater or the use of glucose-lowering medications [33]. Hypertension treatment was recorded based on the use of antihypertensive medications to treat high blood pressure. BMI was calculated by dividing the participant’s weight in kilograms by the square of their height in meters (kg/m^2^).

### 2.5. Statistical Analysis

#### 2.5.1. Comparison of Demographics

Population characteristics were compared based on sex and the trajectory groups of alcohol consumption. Chi-squared tests were used to assess categorical variables, *t*-tests to compare continuous variables between the sexes, and one-way Analysis of Variance (ANOVA) to evaluate differences in continuous variables across trajectory groups.

#### 2.5.2. Trajectory Analysis of Alcohol Consumption

The Growth Mixture Model (GMM) was used to identify longitudinal patterns for alcohol consumption in Phase 1 [35]. The GMM can identify ‘latent classes’, which are hidden groups that the model detects in the data, even though they are not directly observed. Each group is characterized by its own growth parameters: initial status, linear growth, and quadratic growth [35,36]. Covariates in GMM included age, education at baseline in Phase 1, and smoking status at each exam. Both linear and quadratic models were considered to describe different patterns of change in a trajectory over time. Based on the Bayesian Information Criterion (BIC), the use of the quadratic term appeared more appropriate in reflecting curvature variations in drinking behaviors over time. We restricted the number of trajectory groups from one to five based on a combination of statistical model fit, interpretability, and sample size considerations for each cohort- and sex-specific analysis [37]. This resulted in 20 models. The optimal number of groups was selected based on the lowest BIC value, yielding one best-fitting model for each cohort- and sex-specific analysis.

Additional steps were taken to enhance the robustness and interpretability of the results. Because GMM randomly selects initial hyperparameters, we ran 100 iterations per model to obtain stable parameter estimates and reduce random variability [38]. Additionally, we required each identified trajectory group to contain at least 5% of all the study participants to ensure a meaningful group size [39,40]. Lastly, we examined the trajectory groups and combined those that displayed similar alcohol consumption patterns to facilitate a clearer interpretation of further analyses. Sensitivity analyses were conducted to examine whether combining trajectory groups was appropriate (see Supplemental Methods).

#### 2.5.3. Association Analysis of Alcohol Consumption Trajectories with Mortality and CHD

This primary association analysis evaluated the relationship between alcohol consumption trajectories and both all-cause mortality and CHD using sex-specific models combining data from both cohorts. In Phase 2, sex-specific Cox proportional hazards models were conducted to examine the associations between alcohol consumption trajectories and all-cause mortality and incident CHD. The base model quantified the unadjusted association between trajectory groups and an event. Additional covariates, including age, education level, BMI, smoking, hypertension, diabetes, and SBP at baseline of Phase 2, were adjusted for in the multivariable model. We checked the Cox proportional hazards (PH) assumption for all Cox proportional hazards models.

As a secondary association analysis, we conducted the same sex-specific Cox proportional hazards models within each generational cohort, using the same set of covariates, to assess consistency across the two FHS generations. We also conducted multiple sensitivity analyses to assess model robustness, evaluate potential confounding variables, and confirm consistency of associations across cohorts and analytical specifications (Supplemental Materials). All statistical analyses were performed with R version 4.1.1. A two-sided *p* value of less than 0.05 was considered statistically significant.

## 3. Result

In this section, we first described the study sample and alcohol consumption trajectory groups (Phase 1), followed by the association results with mortality and CHD (Phase 2).

### 3.1. Cohort Characteristics

At Phase 1 (Figure 1), our study included 2790 Original cohort participants (mean age at baseline: 63 ± 8; 59.7% women) and 3780 Offspring cohort participants (mean age at baseline: 44 ± 10; 52.1% women) (Table 1). The Original and Offspring cohorts differed significantly in their health profiles (*p* < 0.001), with a mean baseline age difference of 20 years (Table 1). Over half of the Original cohort participants had hypertension (56% in women and 50% in men), while less than one-fourth (17% of the women and 28% of the men) of the Offspring cohort had hypertension. Women in the Original cohort had a higher BMI than those in the Offspring cohort (mean BMI 26.5 vs. 24.7 kg/m^2^). In contrast, men had a similar BMI in both cohorts. Women in the Offspring cohort consumed more alcohol than those in the Original cohort (median consumption 4 g/day vs. 2 g/day, *p* < 0.001). In contrast, men in both cohorts consumed a similar amount of alcohol (median consumption of 14 g/day) (Table 1).

### 3.2. Alcohol Consumption Trajectories

Initially, in the sex- and cohort-specific analyses, we identified five groups of alcohol consumption. Among them, two groups, referred to as Moderate-Decreasing drinking group A and Moderate-Decreasing drinking group B (Supplemental Figure S2), showed similar drinking patterns: starting with moderate alcohol intake (0–28 g/day in men, 0–14 g/day in women) and gradually declining over time. Thus, in the sex- and cohort-specific analyses, these two trajectories were combined into a new group, the Moderate-Decreasing drinking group, to improve interpretability (Supplemental Figure S3).

We also compared alcohol consumption trajectories separately by sex across the Original and Offspring cohorts. Among women, the overall trajectory patterns were broadly similar between the two generational cohorts (Supplemental Figure S3). The Moderate-Decreasing drinking group included participants who began with moderate alcohol intake (approximately 10 g/day, on average) and gradually reduced their intake to less than 5 g/day by the end of Phase 1. The Low-to-None drinking group consisted of women with constant minimal or no alcohol consumption (on average, approximately 0 g/day) throughout the study period. The Inverse-U Pattern drinking group began with a moderate intake of approximately 5 g/day and, around year 7, increased to about 8 g/day in the Original cohort and 10 g/day in the Offspring cohort, then gradually declined to 0 g/day by the end of follow-up. Women in the High-Decreasing drinking group consistently consumed more than 14 g/day throughout the 15-year period, with a slightly lower intake in the Offspring cohort.

Among men, trajectory patterns were broadly similar between the Original and Offspring cohorts, as observed in women. The Moderate-Decreasing drinking group exhibited a gradual decline in alcohol consumption, from approximately 20 g/day at baseline to 12 g/day after 10 years. The Low-to-None drinking group included men who had lightly consumed alcohol (<14 g/day) and non-drinkers. In the Inverse-U Pattern drinking group, the Original cohort participants started at 25 g/day, increasing around years 7–8 to 45 g/day, then decreasing to low-level consumption (<14 g/day) by the end of Phase 1; the Offspring cohort participants began at 30 g/day, peaked at 35 g/day, and also declined to low-level consumption by Phase 1 end. The High-Decreasing drinking group maintained consistently high intake, declining from ~60 g/day to ~45 g/day, with Offspring men averaging slightly higher levels throughout (Supplemental Figure S3).

### 3.3. Association of Alcohol Consumption Trajectories with All-Cause Mortality

**Figure 3:**
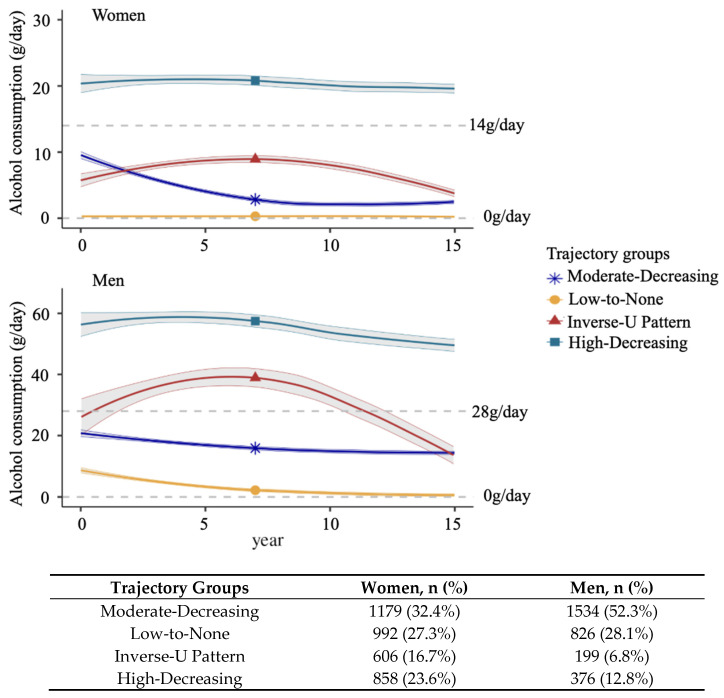
Four trajectory groups of alcohol consumption in sex-specific analysis across the Original and Offspring cohorts. A growth mixture model was applied to identify sex-specific alcohol consumption trajectories. The number and percentage of participants for each trajectory group are presented in the table. The Moderate-Decreasing drinking group included moderate drinkers (<14 g/day for women and <28 g/day for men) who slightly decreased their consumption. The Inverse-U Pattern drinking group comprised participants with varying alcohol intake patterns, while the High-Decreasing drinking group included participants with consistently high intake levels (>28 g/day for women and >40 g/day for men), also showing a decreasing trend.

During an additional median follow-up of 10 years, women have lower mortality rates compared to men (*p* < 0.001): 1124 women (31%; 917 from the Original cohort and 207 from the Offspring cohort) and 1141 men (39%; 790 from the Original cohort and 351 from the Offspring cohort) died from any cause. Mortality was lower in the Offspring cohort compared to the Original cohort (14.7% vs. 61.2%) (Supplemental Table S1). Given the overall similarity in trajectory patterns across cohorts, we conducted the primary association analyses using the combined trajectory groups to maximize statistical power and enhance interpretability (Figure 3). Secondary analyses using cohort-specific groups were performed to compare differences between cohorts. Participants in the Moderate-Decreasing drinking group served as the reference group in all analyses.

**Figure 4:**
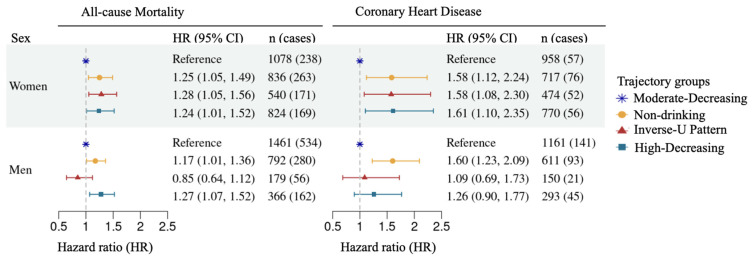
Sex-stratified association analysis between alcohol consumption trajectory groups with all-cause mortality and coronary heart disease. The alcohol consumption groups are described in Figure 3. Cox proportional hazards models were used to examine the association between alcohol consumption trajectory groups and time to all-cause mortality and coronary heart disease. Covariates included age, education level, body mass index, current smoking status, systolic blood pressure, hypertension treatment, and diabetes at the baseline of Phase 2. HR, hazard ratio. 95% CI, 95% confidence interval. *n (cases)*, the total number of participants in a trajectory group (the number of events in this group). The light grey dashed line represents HR = 1 as a reference.

As shown in Supplemental Table S2, women in the Low-to-None drinking group had a lower crude mortality rate (31.6%) compared to the reference group. However, after adjusting for follow-up time using the Cox proportional hazards model, women in the Low-to-None drinking and Inverse-U Pattern drinking groups each exhibited a 24% higher risk of all-cause mortality relative to the reference (Supplemental Table S3). Furthermore, after additionally adjusting for covariates, including age, education level, BMI, smoking status, hypertension, diabetes, and SBP at the baseline of Phase 2, the risk of participants in the Low-to-None drinking group increased by 25% (95% CI = 1.05–1.49, *p* = 0.01), while risk of participants in the Inverse-U Pattern drinking group increased by 28% (95% CI = 1.05–1.56, *p* = 0.01) (Figure 4, Supplemental Table S3). We also found a 22% (95% CI = 1–1.48, *p* = 0.053, considering follow-up time) and 24% (95% CI = 1.01–1.52, *p* = 0.04, adjusting for additional covariates) higher mortality risk for women in the High-Decreasing drinking group compared to the reference group (Supplemental Table S3, Figure S4).

Although men in the Low-to-None drinking group had the lowest crude mortality rate (24.3%) among the four trajectory groups (Supplemental Table S4), Cox proportional hazards modeling revealed a 23% higher risk of mortality compared to the reference group after accounting for follow-up time (95% CI: 1.06–1.42, *p* = 0.005) (Supplemental Figure S4). This elevated risk remained significant, at 17%, after further adjustment for covariates (95% CI: 1.01–1.36, *p* = 0.04) (Figure 4). In addition, men in the High-Decreasing drinking group had a 31% higher risk of all-cause mortality compared to the reference group (95% CI = 1.10–1.56, *p* = 0.003) using the base Cox proportional hazards model and a 27% higher risk (95% CI = 1.07–1.52, *p* = 0.007) after additionally adjusting for covariates. No significant associations were observed for men in the Inverse-U Pattern drinking group compared to the reference group (Figure 4; Supplemental Table S5). The proportional hazards assumption was tested and found to hold in all Cox proportional hazards models.

In secondary analyses, we separately fit sex-specific models in the Original and Offspring cohorts to assess cohort-specific consistency. Our results were generally consistent across generational cohorts within each sex. The combined analysis produced intermediate effect estimates but demonstrated stronger statistical significance and narrower confidence intervals, indicating improved precision and stability of the associations (Supplemental Materials, Supplemental Table S8).

### 3.4. Association of Alcohol Consumption Trajectories with Incident CHD

Over a median follow-up period of 10 years following the Phase 2 baseline, women have a lower incidence rate of CHD compared to men (*p* < 0.001): 263 (7.0%) women (176 from the Original cohort and 87 from the Offspring cohort) and 312 (10.6%) men (155 from the Original cohort and 157 from the Offspring cohort) developed CHD (Supplemental Table S1).

Among women, the crude CHD incidence rates were not significantly different across the trajectory groups without considering follow-up time (*p* = 0.7) (Supplemental Table S2). After considering survival time, women in the Low-to-None and Inverse-U Pattern drinking groups had 57% (95% CI = 1.11–2.21, *p* = 0.01) and 53% (95% CI = 1.05–2.23, *p* = 0.03) higher CHD risk compared to the reference group, and after adjusting for the same set of additional covariates, both risks increased by 58% (Low-to-None drinking group: 95% CI = 1.12–2.24, *p* = 0.01; Inverse-U Pattern drinking group: 95% CI = 1.08–2.30, *p* = 0.02). In the High-Decreasing drinking group, women had a 47% higher risk of developing CHD compared to the reference group (95% CI = 1.02–2.13, *p* = 0.04), and this CHD risk increased to 61% after adjusting for the covariates (95% CI = 1.11–2.35, *p* = 0.01) (Figure 4; Supplemental Table S6).

For men, similar to the observations in women, no significant differences were observed in crude CHD incidence rates across the trajectory groups (*p* = 0.6) (Supplemental Table S3). When incorporating survival time, men in the Low-to-None drinking group had a 60% higher risk of developing CHD compared to the reference group (95% CI = 1.23–2.08, *p* < 0.001), and the risk remained unchanged after adjusting for the same covariates (95% CI = 1.23–2.09, *p* < 0.001). However, men in the Inverse-U Pattern and High-Decreasing drinking groups did not show a significantly different risk of developing CHD compared to the reference before or after adjusting for additional covariates in Cox proportional hazards models (Supplemental Table S7).

Similar to the mortality analyses, results were consistent across generational cohorts within each sex, while the combined analysis yielded intermediate estimates with stronger significance and narrower confidence intervals, reflecting improved precision (Supplemental Materials, Supplemental Table S8).

### 3.5. Sensitivity Analysis to Evaluate Additional Variables

Sensitivity analyses confirmed the robustness of associations between alcohol trajectories and outcomes across alternative model specifications. Results were consistent when varying the reference group, including additional lifestyle and health factors, or adjusting for family structure (Supplemental Table S9 and Figures S5–S8).

## 4. Discussion

This study addresses a critical gap in our understanding of the dynamic nature of alcohol consumption over time and its sex-specific associations with all-cause mortality and CHD. We identified four distinct, sex-specific alcohol consumption trajectories in participants from two longitudinal cohorts in the FHS. Most participants displayed an overall decreasing trend in their alcohol consumption over the 15 years of follow-up. However, the patterns of this decline varied across trajectory groups in both women and men. Using the Moderate-Decreasing drinking group as the reference group, where participants consumed moderate but gradually decreasing amounts of alcohol during the 15-year observation period, we found that women in all three of the other groups displayed significantly higher risks for incident all-cause mortality and CHD. Among men, the Low-to-None drinking group had a higher risk of incident CHD compared to the reference. This group, along with the High-Decreasing drinking group (>28 g/day), also exhibited an increased risk of all-cause mortality. Consistent with prior trajectory-based research, our findings highlight the importance of examining long-term drinking patterns rather than relying on single measures [19,20,21]. Our study extends prior trajectory-based research by focusing on sex-specific trajectories and linking them to adjudicated CHD and mortality outcomes in a large, community-based cohort. Our study showed sex-specific patterns between trajectory groups and the risk of developing all-cause mortality and CHD. Men in our study had higher drinking levels than women across all trajectory groups. Both men and women in the High-Decreasing groups exhibit higher CHD and mortality risks compared to the Moderate-Decreasing group, consistent with recent findings [20,41]. Additionally, in the Inverse-U Pattern drinking groups, both men and women exhibited a trend of increasing followed by decreasing alcohol consumption, while women remained below 14 g/day even at their peak, men exceeded 28 g/day and later reduced their intake to below that level. However, men in the Inverse-U Pattern drinking group showed no significant increase in all-cause mortality and CHD risk compared to the reference group, while women in the Inverse-U Pattern drinking group had a higher risk of both outcomes. These findings are consistent with prior evidence suggesting that alcohol-related cardiovascular effects may follow a biphasic pattern and differ by sex [5,6,12,20,41]. For example, a prior study found that occasional drinking was associated with lower hypertension risk, while frequent drinking was associated with increased risk compared to abstention in women [12]. However, other studies, including large-scale meta-analyses and Mendelian randomization analyses, have challenged the interpretation that low or moderate alcohol consumption has benefits for cardiovascular outcomes and mortality [8,42,43,44]. These inconsistent findings highlight the need for well-designed randomized controlled trials to provide more definitive evidence on this important public health question.

Our findings emphasize the importance of considering sex-specific, long-term drinking trajectories in evaluating cardiovascular and overall health. Although our findings indicate that participants in the Moderate-Decreasing drinking group were associated with a lower risk of all-cause mortality compared to those in the Low-to-None drinking group, differences in baseline characteristics across trajectory groups warrant careful interpretation. Participants in the Low-to-None drinking group had a higher prevalence of preexisting health conditions, such as obesity, diabetes, and hypertension, which are major risk factors for CVD, and may reflect underlying conditions that influence both alcohol consumption patterns and subsequent outcomes. This raises the possibility of reverse causation, whereby individuals reduce or cease alcohol intake due to declining health. Residual confounding and abstainer reference group bias may therefore contribute to the observed associations, particularly given the discrepancy between crude incidence ratios and multivariable-adjusted hazard ratios. Therefore, our findings should not be interpreted as evidence of a protective effect of moderate alcohol consumption.

Our study has several limitations. While we prioritized retaining as much longitudinal data as possible to minimize sample loss, missing data may introduce bias. Additionally, unmeasured covariates or measurement errors may have biased our findings, despite adjusting for multiple factors that influence alcohol consumption patterns. Future research should consider covariates updated at each exam that influence the association between alcohol consumption and health outcomes. Because BMI, blood pressure, and diabetes are major risk factors for CVD and may lie on the causal pathway between alcohol consumption and cardiovascular outcomes, adjustment for these variables may attenuate estimates of the total effect. Accordingly, our fully adjusted models should be interpreted as conditional associations rather than estimates of the total effect of alcohol exposure. We also acknowledge that the self-reported alcohol consumption data and average daily consumption may not fully reflect risks associated with episodic heavy drinking. The longitudinal design of the FHS enables long-term tracking of behavior, helping mitigate biases inherent in self-reporting. In addition, FHS has contributed to large-scale genome-wide association studies (GWAS) [45,46], epigenome-wide association studies (EWAS), and other alcohol-related research with consistently replicated findings [47,48,49]. The variation in exam intervals stems from logistical challenges, including participant availability, resources, and shifting research priorities. While this may affect precision, it is accounted for in statistical models to support reliable conclusions. In addition, FHS Original and Offspring cohorts are predominantly White individuals of European descent with higher levels of education and socioeconomic status, which may limit the generalizability of our findings to more diverse populations [50]. In addition, because participants were primarily related family members and had relatively high educational attainment [24], the generalizability of these results to unrelated populations or to different geographic and sociodemographic contexts warrants careful consideration. Therefore, future studies should include a more diverse group of individuals to enhance the applicability of the findings.

Despite its limitations, this study has several advantages. First, over 15 years of alcohol consumption data provide a rich resource for characterizing long-term drinking patterns. Second, the inclusion of two generational cohorts may capture temporal changes in socioeconomic conditions, medical practices, and lifestyle behaviors, particularly in relation to alcohol use and CVD prevention and treatment. While some cohort-specific differences were observed in trajectory patterns and associations, the overall direction of associations was consistent. Therefore, we combined the cohorts to increase sample size and precision, which strengthened the robustness and interpretability of our findings.

## 5. Conclusions

In summary, this study identified sex-specific alcohol consumption trajectories across two FHS generations. Our findings suggest that sustained low to moderate drinking was observationally associated with lower risks of mortality and CHD in both women and men, compared to high-level or fluctuating patterns. However, given the observational design, our findings cannot establish causality and may be influenced by residual confounding or reverse causation. While our findings highlight the importance of considering long-term drinking patterns in public health research, they should not be interpreted as evidence that moderate alcohol consumption reduces mortality risk or prevents CHD.

## Figures and Tables

**Table 1:** Baseline characteristics of the study samples.

Variable ^1^	Original Cohort		Offspring Cohort	
	Men (n = 1123)	Women (n = 1667)	Men (n = 1812)	Women (n = 1968)
Age, years	63 (7)	64 (8)	44 (10)	44 (10)
BMI, kg/m^2^	26.9 (3.4)	26.46 (4.5)	26.76 (3.6)	24.66 (4.7)
DBP, mmHg	84.5 (12.1)	83.63 (12.1)	82.03 (10.6)	76.07 (10.6)
SBP, mmHg	141.6 (21.9)	144.91 (25.7)	127.37 (17.3)	119.49 (18.5)
TC, mg/dL	225.1 (38.2)	227.1 (43.5)	209.02 (38.0)	203.85 (40.9)
HDL, mg/dL ^2^	NA	NA	43.20 (11.8)	55.55 (14.9)
Education, *n* (%)				
No high school	433 (38.6%)	611 (36.7%)	131 (7.2%)	110 (5.6%)
High school	344 (30.6%)	546 (32.8%)	521 (28.8%)	679 (34.5%)
Some college	148 (13.2%)	353 (21.2%)	441 (24.3%)	635 (32.3%)
College or above	198 (17.6%)	157 (9.4%)	719 (39.7%)	544 (27.6%)
Hypertension treatment, *n* (%)	160 (14.3%)	371 (22.3%)	194 (10.7%)	180 (9.1%)
Lipid treatment, *n* (%)	13 (1.2%)	38 (2.3%)	21 (1.2%)	10 (0.5%)
Current diabetes, *n* (%)	4 (9.8%)	4 (6.6%)	60 (3.4%)	36 (1.9%)
Obesity, *n* (%)	176 (15.7%)	312 (18.8%)	297 (16.5%)	238 (12.1%)
Hypertension, *n* (%) ^3^	566 (50.4%)	929 (55.7%)	506 (27.9%)	343 (17.4%)
Alcohol consumption, g/day ^4^	14 (4, 23)	2 (0, 12)	14 (4, 22)	4 (0, 14)

^1^ Values were presented as mean (SD) for continuous variables and count (percent) for category variables. ^2^ HDL information was not available for the Original cohort at exam 12. ^3^ Hypertension was defined as the use of hypertension treatment, SBP ≥ 130 mmHg, or diastolic blood pressure (DBP) ≥ 80 mmHg. ^4^ Median (IQR) was reported. Note: All variables showed significant differences across the four groups (all *p* < 0.001).

## Data Availability

The data could be requested through an application to the FHS (https://www.framinghamheartstudy.org/fhs-for-researchers, accessed on 31 January 2026).

## Supplementary Materials

The following supporting information can be downloaded at: https://www.mdpi.com/article/10.3390/nu18050849/s1, Supplemental Methods; Supplemental Results; Supplemental Table S1. Characteristics of the study participants at baseline of Phase 2; Supplemental Table S2. Women: Characteristics of the study sample at baseline of Phase 2, stratified by alcohol consumption trajectory groups; Supplemental Table S3. Women: Association between alcohol consumption trajectories and all-cause mortality during a 10-year follow-up period; Supplemental Table S4. Men: Characteristics of the study sample at baseline of Phase 2 stratified by alcohol consumption trajectory groups; Supplemental Table S5. Men: Longitudinal association between alcohol consumption trajectories and all-cause mortality during a 10-year follow-up period; Supplemental Table S6. Women: Longitudinal association between alcohol consumption trajectories and incident CHD during a 10-year follow-up period; Supplemental Table S7. Men: Longitudinal association between alcohol consumption trajectories and coronary heart disease (CHD) during a 10-year follow-up period; Supplementary Table S8. The Sex-specific association analysis between alcohol consumption trajectory groups with all-cause mortality and coronary heart disease (CHD) for participants in the Original cohort, the offspring cohort, and the combined sample during a 10-year follow-up period; Supplementary Table S9. The Sex-stratified association analysis between 5 unmerged alcohol consumption trajectory groups with CHD and all-cause mortality during a 10-year follow-up period; Supplemental Figure S1: Distribution of alcohol consumption; Supplemental Figure S2. Five trajectory groups of alcohol consumption in sex- and cohort-specific analysis. Supplemental Figure S3. Four trajectory groups of alcohol consumption in sex- and cohort-specific analysis. Supplemental Figure S4. Unadjusted sex-stratified association analysis between alcohol consumption trajectory groups with all-cause mortality and incident CHD. Supplementary Figure S5. Sex-stratified association analysis of alcohol consumption trajectory groups with all-cause mortality and incident CHD, comparing models with individual covariates versus those adjusted using the ASRS. Supplementary Figure S6. Sex-stratified association analyses of alcohol consumption trajectory groups with all-cause mortality and incident CHD, comparing models with individual covariates versus those adjusted the FRS. Supplementary Figure S7. Sex-stratified association analyses of alcohol consumption trajectory groups with all-cause mortality and incident CHD, comparing the models adjusted for primary individual covariates with those adjusted for additional covariates. Supplementary Figure S8. Sex-stratified association analyses of alcohol consumption trajectory groups with all-cause mortality and incident CHD, adjusting for family ID [51,52].

## References

[1] (2018). Collaborators, Global Burden of Disease. Alcohol use and burden for 195 countries and territories, 1990-2016: A systematic analysis for the Global Burden of Disease Study 2016. Lancet.

[2] Greaves L. (2020). Missing in Action: Sex and Gender in Substance Use Research. Int. J. Environ. Res. Public Health.

[3] Mostofsky E., Chahal H.S., Mukamal K.J., Rimm E.B., Mittleman M.A. (2016). Alcohol and Immediate Risk of Cardiovascular Events: A Systematic Review and Dose-Response Meta-Analysis. Circulation.

[4] Hemsing N., Greaves L. (2020). Gender Norms, Roles and Relations and Cannabis-Use Patterns: A Scoping Review. Int. J. Environ. Res. Public Health.

[5] Chiva-Blanch G., Badimon L. (2019). Benefits and Risks of Moderate Alcohol Consumption on Cardiovascular Disease: Current Findings and Controversies. Nutrients.

[6] Colpani V., Baena C.P., Jaspers L., van Dijk G.M., Farajzadegan Z., Dhana K., Tielemans M.J., Voortman T., Freak-Poli R., Veloso G.G.V. (2018). Lifestyle factors, cardiovascular disease and all-cause mortality in middle-aged and elderly women: A systematic review and meta-analysis. Eur. J. Epidemiol..

[7] Roerecke M., Rehm J. (2014). Alcohol consumption, drinking patterns, and ischemic heart disease: A narrative review of meta-analyses and a systematic review and meta-analysis of the impact of heavy drinking occasions on risk for moderate drinkers. BMC Med..

[8] Zhao J., Stockwell T., Naimi T., Churchill S., Clay J., Sherk A. (2023). Association Between Daily Alcohol Intake and Risk of All-Cause Mortality: A Systematic Review and Meta-analyses. JAMA Netw. Open.

[9] Ronksley P.E., Brien S.E., Turner B.J., Mukamal K.J., Ghali W.A. (2011). Association of alcohol consumption with selected cardiovascular disease outcomes: A systematic review and meta-analysis. BMJ.

[10] Mumenthaler M.S., Taylor J.L., O’Hara R., Yesavage J.A. (1999). Gender differences in moderate drinking effects. Alcohol. Res. Health.

[11] Vatsalya V., Liaquat H.B., Ghosh K., Mokshagundam S.P., McClain C.J. (2016). A Review on the Sex Differences in Organ and System Pathology with Alcohol Drinking. Curr. Drug Abuse Rev..

[12] Vorobelova L., Falbova D., Candrakova Cernanova V. (2022). Contribution of environmental factors and female reproductive history to hypertension and obesity incidence in later life. Ann. Hum. Biol..

[13] Lee K. (2018). Sex-Specific Associations of Risk-Based Alcohol Drinking Level with Cardiovascular Risk Factors and the 10-Year Cardiovascular Disease Risk Scores. Alcohol. Clin. Exp. Res..

[14] Simon J., Fung K., Kolossváry M., Sanghvi M.M., Aung N., Paiva J.M., Lukaschuk E., Carapella V., Merkely B., Bittencourt M.S. (2021). Sex-specific associations between alcohol consumption, cardiac morphology, and function as assessed by magnetic resonance imaging: Insights form the UK Biobank Population Study. Eur. Heart J. Cardiovasc. Imaging.

[15] Naimi T.S., Stockwell T., Saitz R., Chikritzhs T. (2017). Selection bias and relationships between alcohol consumption and mortality. Addiction.

[16] Georgescu O.S., Martin L., Târtea G.C., Rotaru-Zavaleanu A.D., Dinescu S.N., Vasile R.C., Gresita A., Gheorman V., Aldea M., Dinescu V.C. (2024). Alcohol Consumption and Cardiovascular Disease: A Narrative Review of Evolving Perspectives and Long-Term Implications. Life.

[17] Khamis A.A., Salleh S.Z., Ab Karim M.S., Mohd Rom N.A., Janasekaran S., Idris A., Abd Rashid R.B. (2022). Alcohol Consumption Patterns: A Systematic Review of Demographic and Sociocultural Influencing Factors. Int. J. Environ. Res. Public Health.

[18] Millwood I.Y., Walters R.G., Mei X.W., Guo Y., Yang L., Bian Z., Bennett D.A., Chen Y., Dong C., Hu R. (2019). Conventional and genetic evidence on alcohol and vascular disease aetiology: A prospective study of 500 000 men and women in China. Lancet.

[19] Llaha F., Licaj I., Sharashova E., Benjaminsen Borch K., Lukic M. (2025). Alcohol consumption trajectories and associated factors in adult women: The Norwegian Women and Cancer study. Alcohol Alcohol..

[20] Bassett J.K., Peng Y., MacInnis R.J., Hodge A.M., Lynch B.M., Room R., Giles G.G., Milne R.L., Jayasekara H. (2025). Alcohol consumption trajectories over the life course and all-cause and disease-specific mortality: The Melbourne Collaborative Cohort Study. Int. J. Epidemiol..

[21] Ranker L.R., Ross C.S., Rudolph A.E., Weuve J., Xuan Z. (2023). Identifying and describing trajectories of alcohol use frequency and binge drinking frequency among those aged 15–30 years in a national cohort of US adolescents: A group-based trajectory modeling approach. Addiction.

[22] Dawber T.R., Meadors G.F., Moore F.E. (1951). Epidemiological approaches to heart disease: The Framingham Study. Am. J. Public Health Nations Health.

[23] Feinleib M., Kannel W.B., Garrison R.J., McNamara P.M., Castelli W.P. (1975). The Framingham Offspring Study. Design and preliminary data. Prev. Med..

[24] Hammond M.M., Zhang Y., Pathiravasan C.H., Lin H., Sardana M., Trinquart L., Benjamin E.J., Borrelli B., Manders E.S., Fusco K. (2022). Relations Between BMI Trajectories and Habitual Physical Activity Measured by a Smartwatch in the Electronic Cohort of the Framingham Heart Study: Cohort Study. JMIR Cardio.

[25] Whitlock G., Lewington S., Sherliker P., Clarke R., Emberson J., Halsey J., Qizilbash N., Collins R., Peto R., Prospective Studies Collaboration (2009). Body-mass index and cause-specific mortality in 900 000 adults: Collaborative analyses of 57 prospective studies. Lancet.

[26] Di Angelantonio E., Bhupathiraju S.N., Wormser D., Gao P., Kaptoge S., Berrington de Gonzalez A., Cairns B.J., Huxley R., Jackson C.L., Global BMI Mortality Collaboration (2016). Body-mass index and all-cause mortality: Individual-participant-data meta-analysis of 239 prospective studies in four continents. Lancet.

[27] Sun X., Ho J.E., Gao H., Evangelou E., Yao C., Huan T., Hwang S.J., Courchesne P., Larson M.G., Levy D. (2021). Associations of Alcohol Consumption with Cardiovascular Disease-Related Proteomic Biomarkers: The Framingham Heart Study. J. Nutr..

[28] Liu C., Marioni R.E., Hedman Å.K., Pfeiffer L., Tsai P.C., Reynolds L.M., Just A.C., Duan Q., Boer C.G., Tanaka T. (2018). A DNA methylation biomarker of alcohol consumption. Mol. Psychiatry.

[29] Box G.E.P., Cox D.R. (1964). An Analysis of Transformations. J. R. Stat. Soc. Ser. B (Methodol.).

[30] Culleton B.F., Larson M.G., Wilson P.W., Evans J.C., Parfrey P.S., Levy D. (1999). Cardiovascular disease and mortality in a community-based cohort with mild renal insufficiency. Kidney Int..

[31] Cupples L.A., D’Agostino R.B., Anderson K., Kannel W.B. (1988). Comparison of baseline and repeated measure covariate techniques in the Framingham Heart Study. Stat. Med..

[32] Kannel W.B., McGee D., Gordon T. (1976). A general cardiovascular risk profile: The Framingham Study. Am. J. Cardiol..

[33] Ho J.E., Larson M.G., Ghorbani A., Cheng S., Coglianese E.E., Vasan R.S., Wang T.J. (2014). Long-term cardiovascular risks associated with an elevated heart rate: The Framingham Heart Study. J. Am. Heart Assoc..

[34] Kannel W., Wolf P., Garrison R. (1988). The Framingham Heart Study, Section 34: An Epidemiological Investigation of Cardiovascular Disease: Some Risk Factors Related to the Annual Incidence of Cardiovascular Disease and Death in Pooled Repeated Biennial Measurements: 30-Year Follow-Up. Lung, and Blood Institute. https://ntrl.ntis.gov/NTRL/dashboard/searchResults/titleDetail/PB87177499.xhtml.

[35] Jo B., Findling R.L., Wang C.P., Hastie T.J., Youngstrom E.A., Arnold L.E., Fristad M.A., Horwitz S.M. (2017). Targeted use of growth mixture modeling: A learning perspective. Stat. Med..

[36] Wardenaar K.J. (2020). Latent Class Growth Analysis and Growth Mixture Modeling using R: A tutorial for two R-packages and a comparison with Mplus. PsyArXiv.

[37] Rahman F., Yin X., Larson M.G., Ellinor P.T., Lubitz S.A., Vasan R.S., McManus D.D., Magnani J.W., Benjamin E.J. (2016). Trajectories of Risk Factors and Risk of New-Onset Atrial Fibrillation in the Framingham Heart Study. Hypertension.

[38] Hipp J.R., Bauer D.J. (2006). Local solutions in the estimation of growth mixture models. Psychol. Methods.

[39] Sagaon-Teyssier L., Mabire X., Laguette V., Fressard L., Suzan-Monti M., Rojas Castro D., Hall N., Capitant C., Meyer L., Chidiac C. (2018). A Group-Based Trajectory Model for Changes in Pre-Exposure Prophylaxis and Condom Use Among Men Who Have Sex with Men Participating in the ANRS IPERGAY Trial. AIDS Patient Care STDs.

[40] Dillon P., Stewart D., Smith S.M., Gallagher P., Cousins G. (2018). Group-Based Trajectory Models: Assessing Adherence to Antihypertensive Medication in Older Adults in a Community Pharmacy Setting. Clin. Pharmacol. Ther..

[41] Qiu W., Cai A., Li L., Feng Y. (2022). Longitudinal Trajectories of Alcohol Consumption with All-Cause Mortality, Hypertension, and Blood Pressure Change: Results from CHNS Cohort, 1993–2015. Nutrients.

[42] Carr S., Bryazka D., McLaughlin S.A., Zheng P., Bahadursingh S., Aravkin A.Y., Hay S.I., Lawlor H.R., Mullany E.C., Murray C.J.L. (2024). A burden of proof study on alcohol consumption and ischemic heart disease. Nat. Commun..

[43] Piepoli M.F., Abreu A., Albus C., Ambrosetti M., Brotons C., Catapano A.L., Corra U., Cosyns B., Deaton C., Graham I. (2020). Update on cardiovascular prevention in clinical practice: A position paper of the European Association of Preventive Cardiology of the European Society of Cardiology. Eur. J. Prev. Cardiol..

[44] Holmes M.V., Dale C.E., Zuccolo L., Silverwood R.J., Guo Y., Ye Z., Prieto-Merino D., Dehghan A., Trompet S., Wong A. (2014). Association between alcohol and cardiovascular disease: Mendelian randomisation analysis based on individual participant data. BMJ.

[45] Saunders G.R.B., Wang X., Chen F., Jang S.K., Liu M., Wang C., Gao S., Jiang Y., Khunsriraksakul C., Otto J.M. (2022). Genetic diversity fuels gene discovery for tobacco and alcohol use. Nature.

[46] Schumann G., Liu C., O’Reilly P., Gao H., Song P., Xu B., Ruggeri B., Amin N., Jia T., Preis S. (2016). KLB is associated with alcohol drinking, and its gene product beta-Klotho is necessary for FGF21 regulation of alcohol preference. Proc. Natl. Acad. Sci. USA.

[47] Wang M., Li Y., Lai M., Nannini D.R., Hou L., Joehanes R., Huan T., Levy D., Ma J., Liu C. (2023). Alcohol consumption and epigenetic age acceleration across human adulthood. Aging.

[48] Bui H., Keshawarz A., Wang M., Lee M., Ratliff S.M., Lin L., Birditt K.S., Faul J.D., Peters A., Gieger C. (2024). Association analysis between an epigenetic alcohol risk score and blood pressure. Clin. Epigenet..

[49] Yousefi P.D., Richmond R., Langdon R., Ness A., Liu C., Levy D., Relton C., Suderman M., Zuccolo L. (2019). Validation and characterisation of a DNA methylation alcohol biomarker across the life course. Clin. Epigenet..

[50] Loucks E.B., Lynch J.W., Pilote L., Fuhrer R., Almeida N.D., Richard H., Agha G., Murabito J.M., Benjamin E.J. (2009). Life-course socioeconomic position and incidence of coronary heart disease: The Framingham Offspring Study. Am. J. Epidemiol..

[51] Tan Z.S., Spartano N.L., Beiser A.S., DeCarli C., Auerbach S.H., Vasan R.S., Seshadri S. (2017). Physical Activity, Brain Volume, and Dementia Risk: The Framingham Study. J. Gerontol. A Biol. Sci. Med. Sci..

[52] Wang X., Pathiravasan C.H., Zhang Y., Trinquart L., Borrelli B., Spartano N.L., Lin H., Nowak C., Kheterpal V., Benjamin E.J. (2023). Association of Depressive Symptom Trajectory With Physical Activity Collected by mHealth Devices in the Electronic Framingham Heart Study: Cohort Study. JMIR Ment Health.

