# Supplementary material for: Sex-Specific Fifteen-Year Alcohol Consumption Trajectories and Their Association with Cardiovascular Events and Mortality: The Framingham Heart Study
Source: Nutrients. 2026 Mar 5;18(5):849. doi: 10.3390/nu18050849 (PMC12986612; doi:10.3390/nu18050849)
Supplement: Supplementary file 1 [file nutrients-18-00849-s001.zip › Exam11.6_consent_eng.pdf]

Framingham Heart Study

Offspring Exam 11, Omni 1 Exam 6

Research Consent Form

Welcome Back to the Framingham Heart Study

Together we are helping to fight heart disease and other major diseases and health conditions through research.

Basic Information

Title of Project: Framingham Heart Study

IRB Number: H-32132

Sponsor: National Heart, Lung, and Blood Institute (NHLBI), National Institutes of Health (NIH)

Principal Investigator(s): Donald Lloyd-Jones, MD, ScM
Framingham Heart Study
73 Mount Wayte Ave
Framingham, MA 01702

Joanne Murabito, MD
Framingham Heart Study
73 Mount Wayte Ave
Framingham, MA 01702

Study Center Email: FHS@bu.edu

Study Center Phone Number: (508)- 872-6562 or (800) 854-7582)

Why is the research being done? (Overview and Purpose)

The Framingham Heart Study is a long-term research study. The purpose of the study is:

(1) To help understand how heart and blood vessel diseases, lung and blood diseases, stroke, memory loss, cancer and other major diseases and health conditions develop: and
(2) To examine DNA and its relationship to the risks of developing these diseases and other health conditions.

The research examination that will be conducted as part of this study is not clinical care. The tests are for research purposes only. We do not provide medical services. This research examination does not take the place of medical care by your own health care provider.

BMC and BU Medical Campus IRB
IRB NUMBER: H-32132
IRB APPROVAL DATE: 12/02/2025

V29En

About Your Consent

Please read this research consent form carefully. It tells you important information about the research study. Taking part in a research study is voluntary. The decision to take part in all or any part of the research exam is entirely up to you. If you choose to take part, you can decide to stop at any time. Your decision will be honored and respected. There will be no penalty for you if you decide to stop or not to take part.

What Will Happen in This Research Study?

You will need to fast for 10 hours before you come to the study appointment for the blood draw. You can continue to drink water while fasting and take your usual medication on the morning of your visit. Most medication can be taken while fasting, but you should consult with your health care providers if you have questions or concerns.

Where Will Your Research Examination Take Place?

We are offering several options for how participants may complete the exam. Participants may choose to participate in:

- In-person exam at FHS Research Center (73 Mount Wayte Ave, Framingham MA), over 1 or multiple sessions (full exam will take around 4 hours to complete).
- Televisit Exam with FHS Staff via phone or online videoconference (Zoom).
- Hybrid Exam consisting of a televisit via phone or online videoconference (Zoom) and a shorter in-person exam at the FHS Research Center.
- Offsite exam in your home or other residence.

Note for televisit and offsite exams: The televisit and offsite visit will have the option of a third-party mobile phlebotomy unit, which would come to the participant’s home or other residence. The mobile unit would have the ability to collect a blood sample, ECG, blood pressure, hip and waist measurements, height and weight. Third-party mobile phlebotomy units are administered through ExamOne, a Quest Diagnostics company. Blood specimen testing will be conducted by Framingham Heart Study Staff.

Only exam components that may be completed remotely will be included during the televisit. Some components that require in-person assessment may be conducted during visits to the Research Center or offsite appointments in which FHS staff or ExamOne staff visit you in your home or other residence.

Some of the study components described in this section below may not be administered during televisit exams, due to the need to take certain measurements in person. Also, some of the study components may not be administered during televisit or offsite examinations taking place in your home or care facility, due to large equipment that cannot be transported outside of the FHS Research Center. Offsite examinations will also require a smaller blood sample collection than blood collection in the FHS Research Center due to the time sensitivity of blood processing.

BMC and BU Medical Campus IRB
IRB NUMBER: H-32132
IRB APPROVAL DATE: 12/02/2025

As before in previous FHS Exams for your cohorts, we will again:

- Draw a sample of blood for genetic and laboratory tests to better understand risk factors for heart disease and other diseases under investigation (for example, the amount and function of different types of cholesterol in your blood).
    - For visits at the FHS Research Center, the total blood draw will be up to 80 mL, which is about 5.4 tablespoons.
    - For offsite visits with FHS staff, the total blood draw will be up to 34 mL, which is about 2.3 tablespoons
    - For ExamOne visits, the total blood draw will be up to 22 mL, is which is about 1.5 tablespoons
- Measure your height, weight, waist, and hips.
- Collect a urine sample (during in person visits to the FHS Research Center only)
- Administer questionnaires related to your health and behaviors.
- Complete an electrocardiogram (ECG).
- Record your blood pressure.
- Measure your hand grip strength.
- Observe you walk a short distance.
- Administer a blood questionnaire.
- Update your medical history information.
- Ask you to sign an authorization form to allow FHS to obtain copies of your **medical records**. The release form gives us permission to obtain these records now and after your death unless you revoke permission.
- Contact you later by mail, email, or by phone (call or text) to obtain additional information. You may also be invited to complete **annual medical history updates**.
- Contact you in person, by mail, email, or phone (call or text) to invite you to participate in **future additional FHS-related studies**. You may also be invited to return for another examination in the future. As always, your participation is voluntary.
- In the event of a stroke while you are an FHS participant, you will be invited to research study visits to occur at the time of this event and on a limited schedule afterwards. A neurologic evaluation will be performed, and your memory, thinking, and activities of daily living will be examined.

Surveys

We will also be asking you to complete questionnaires about your physical function, diet, exercise, memory and mood, and lifestyle habits, including whether you smoke or use alcohol. Some of the questionnaires you have seen before, and others will be new to you.

Some of your responses may be recorded using a digital audio recorder. We use recordings to make sure that your responses are accurately documented for quality control purposes.

BMC and BU Medical Campus IRB
IRB NUMBER: H-32132
IRB APPROVAL DATE: 12/02/2025

Additional Medical Record Release Using Medicare/Medicaid Insurance Information

You will be asked to provide your Medicare and/or Medicaid Beneficiary Identifiers on the participant contact information sheet that is retained by the research team at Boston University Chobanian and Avedisian School of Medicine using secure systems. The Medicare and/or Medicaid Beneficiary Numbers are being collected to obtain information from the Centers for Medicare and Medicaid Services (CMS). This information will help us understand the bigger picture of your medical history and medical service usage, and how use of health care may change as people age. Sharing these Identifiers will allow us to link your FHS data with CMS data using your name and other information and share the data with other researchers using a virtual data enclave called LINKAGE managed by the National Institute on Aging (NIA). Sharing your Medicare and/or Medicaid Beneficiary Identifiers is strictly optional and is not required for participation in the study.

There are also research activities that will be NEW to your participant cohort:

Medication Self-Administration and Self-Awareness (MSA) Study: In this study, you will be asked to complete a standardized test designed to assess how you understand and take medications when given instructions [12 minutes]. In the written part of this test, you will be asked to read medication instructions and then fill in the blanks on a schedule to indicate when you will take them. In the manual part of this test, we will ask you to distribute pretend medications in a divided pillbox according to the medication instructions. You will complete a brief written rating at the end to indicate how you did on this test [1 minute]. You will also be asked to complete a timed, 7-minute survey to measure your ability to read, understand, and act on health care information. If your spouse is also a FHS participant and attends the exam with you, we will ask them to complete a short questionnaire assessing the approximate amount of time they spend assisting you in your daily life [5 mins]. We believe participation in this entire activity will take no longer than 20 minutes. **Participation in this study is only possible for in-person visits to the FHS Research Center.**

What risks can I expect? There are no known risks to taking part in this study. Completion of assessments may cause mild frustration in those who find assessments difficult.

Overall Examination Risks and Discomforts

General Risks related to FHS: The FHS research exam is time consuming and repetitive. Other discomforts may include headache, feeling hungry due to fasting, fatigue, and chill during the visit. Also, because of the blood draw, minimal bruising, pain, bleeding, lightheadedness/fainting, or, in rare circumstances, infection may occur.

Risks specific to new ancillary studies' activities are discussed in the section above.

Unknown risk: There may also be some risks that we are unable to determine at this time.

We do not expect any risk of injury as a result of your participation in the study. However, first aid will be available.

BMC and BU Medical Campus IRB
IRB NUMBER: H-32132
IRB APPROVAL DATE: 12/02/2025

Genetic Studies

You may have already provided consent in the past for the collection of biological samples (for example, white blood cells) for DNA research or in the creation of Induced Pluripotent Stem Cells (iPS cells) to study specific tissue types. We plan to continue to do genetic research on the DNA from your biological samples. DNA is the material that makes up your genes. Genes are passed from parent to child. All living things are made of cells. Genes are the part of cells that contain the instructions which tell our bodies how to grow and work and determine physical characteristics such as hair and eye color.

We will use white blood cells from your blood sample for research. These cells can be studied as they are, or we can change them in the lab to work like cells from other parts of your body, such as heart cells, liver cells, or brain cells (iPS cells). We call these changed cells “stem cells.”

We study these cells to learn what causes diseases and how to treat them. Your cells will be stored at Boston University and may be shared with other researchers. They might be used in animal studies or to develop new treatments or tests for diseases.

Scientists may change the genes in your cells for research, but they will never use them to create a whole human being. If researchers develop products or treatments using your cells, you will not receive money from this.

Your cells may be used for many years in different research studies, including ones we haven’t planned yet.

You may choose to participate in non-genetic FHS research without choosing to participate in genetic research. Later in the form you are asked to specifically indicate if you choose to participate in genetic research.

How will I learn the results of the study?

Core Framingham Heart Study: The main way results of research from this study are reported is in scientific publications and presentations at scientific meetings. Summary findings are also sometimes described in our newsletters and on our study website.

We will also report some routine research test measurements to your and/or your health care provider at the time of the exam or after your visit. These may include, for example, blood pressure and cholesterol.

In some cases, if we determine it to be appropriate, we may report to you and/or your health care provider research findings as they relate to you, if you give permission. This information, if it is reported, might be reported long after your visit for a number of reasons.

As an example, it might take years of work to analyze information and arrive at research findings, possibly using newly developed scientific methods.

Our genetic research might generate findings that could be relevant to you and possibly your family members, such as information about a particular genetic variant that might put you at risk

BMC and BU Medical Campus IRB
IRB NUMBER: H-32132
IRB APPROVAL DATE: 12/02/2025

of a serious health condition. At this time, we believe that most of the genetic research findings do not have medical importance to individuals, but the field of genetics is changing rapidly.

We currently do not have specific plans to contact you or your health care provider about genetic or non-genetic findings other than some routine research test measurements. In general, we cannot commit to providing any other research findings to you. In determining whether we share additional research information with you, we will take into account a number of considerations on a case-by-case basis. These might include whether the findings were based on tests that are clinically acceptable, accurate and reliable, whether the findings reveal a significant risk of a serious health condition, whether there is, at the relevant time, a recognized treatment or prevention intervention or other available actions that have the potential to change the clinical course of the health condition, whether reporting or not reporting the results is likely to increase the risk of harm to you, and other relevant factors we might not be able to predict at this time. In the cases when genetic research findings are reported to you, a study investigator and genetic counselor will contact you to confirm your continued interest in hearing about genetic research results. If you confirm your interest, the study staff will inform you of the research results and recommend next steps such as obtaining confirmatory clinical testing and speaking to your personal health care provider.

Research test measurements and findings are not the same as clinical results. As such, our research examination is not necessarily performed by individuals with clinical training and qualifications, and many parts of the examination do not meet the standards for certified clinical testing. For these reasons, **our research tests should not be relied on to make any diagnosis, treatment, or health planning decisions. We do not provide health care or give medical advice or genetic testing or provide counseling**. If you or your health care provider decide that follow up tests or treatments are necessary, then you (or a third party such as health insurance carrier or Medicare) will be responsible for the cost.

How are my samples and information shared with other researchers?

Samples and information will be kept indefinitely. If you agree, your data and donated blood, blood cells, urine, and any other specimens may be shared with other researchers. These include academic, non-profit, and for-profit entities, including but not limited to hospitals, universities, cell/tissue storage banks and repositories, databanks, and data repositories and businesses, whether for related or unrelated research studies. The cell/tissue storage banks and repositories, databanks, and data repositories, include but are not limited to NIH repositories dbGaP and BioLINCC. Internal and external researchers may request data and materials for research. The repositories have standard operating procedures to protect your confidentiality. Your data and samples will not be labeled with your name or other direct personal identifiers, only a code.

Your name and other direct personal identifiers will not be shared with these entities.

You have the right to refuse to allow your data and samples to be used or shared for further research. Please check the appropriate box in the section below.

BMC and BU Medical Campus IRB
IRB NUMBER: H-32132
IRB APPROVAL DATE: 12/02/2025

If you give your permission to allow your data and biological samples to be used or shared for further research, you may withdraw your permission at any time by contacting the FHS investigators. However, if your data or samples have already been released to other researchers, we will not be able to instruct the other researchers to stop using them, to destroy them or products made from them. Your data and samples will not include your name or other identifiers.

What risks can I expect? (Risks and Discomforts)

General risks and individual risks related to new activities are discussed above.

Participating in genetic research could have a negative impact on you, your family, and your loved ones. The genetic studies might result in research findings that relate to your risk of a serious health condition or other genetic information that we might consider to be appropriate to reports to you and your health care provider, if you wish us to report them (see below). This could present you with some difficult decisions regarding the available information and the disease risks you and your family members might face. Knowledge of genetic research findings can provoke anxiety and influence decisions regarding marriage, family planning, and other matters. There is a potential risk that your genetic information could be used to your disadvantage. For example, if genetic research findings suggest a serious health problem, that could be used to make it harder for you to get or keep a job or insurance. Both Massachusetts state laws and federal laws, particularly the Genetic Information Nondiscrimination Act (GINA), generally make it illegal for health insurance companies, group health plans, and most employers to discriminate against you based on your genetic information. These laws will generally protect you in the following ways:

1. Health insurance companies and group health plans may not request your genetic information that we get from this research.
2. Health insurance companies and group health plans may not use your genetic information when making decisions regarding your eligibility or premiums.
3. Massachusetts employers with 6 or more employees (or 15 or more employees in other states, under GINA) may not use your genetic information that we get from this research when making a decision to hire, promote, or fire you or when setting the terms of your employment.

Be aware that neither Massachusetts law nor GINA protects you against genetic discrimination by companies that sell life insurance, disability insurance, or long-term care insurance.

Thus, life insurance, disability insurance, and long-term care insurance companies may legally ask whether you have had genetic testing and deny coverage for refusal to answer this question.

What are the possible benefits from being in this research study? (Potential Benefits)

While you will not receive any direct benefit as a result of your participation in this study, we hope that this study will help us better understand what causes heart disease and other diseases and conditions and how to better prevent and treat them.

What are the costs of taking part in the study? (Costs)

Costs that you may incur on the day of your participation include, but are not limited to, loss of work and transportation costs (gas, tolls, etc).

Payment

As a token of our appreciation for participating in this exam cycle, you will receive a gift card at the end of your visit with Framingham Heart Study. Below is a grid outlining the study activities you may participate in:

Research Activities	Appreciation Amount for Completing in Person at Framingham Research Center	Appreciation Amount for Completing via televisit or offsite visit
Core Exam, Questionnaires, Surveys, EKG, Blood Pressure, Blood Draw and Health Interview	$125	$100 (Some blood draw components excluded from offsite activities)
Research Stations:<br>MSA Study	$25	N/A
**Total Appreciation Amount Available:**	**$150**	**$100**

In addition to the studies activities described above, you may also have the opportunity to take part in further, optional ancillary studies, which require another consent form and provide an additional appreciation payment. If you choose to learn more about those ancillary studies, we will consolidate all payments from all studies you participate in at the end of the visit

In compliance with federal and state laws, if the total amount of payments is more than $600 in a calendar year you are required to provide us with your Social Security Number or Individual Taxpayer Identification Number to receive this payment.

The research may also lead to the development of drugs, tests, or procedures that might have commercial value. You will not receive financial compensation if products are developed from the research.

How is my information protected? (Confidentiality)

Data and Samples Storage: We will store your information in ways we think are secure. We will store biological samples taken from your body (such as urine, blood, or tissue). We label your samples and information with a code, and we keep the key to the code in a password-protected database. Only approved staff are given the password. We will store paper files in locked filing cabinets and secured rooms. We will store electronic files in computer systems with password protection and encryption.

Only the people listed later in this section will be given access to your information. However, we cannot guarantee complete confidentiality.

Protection of information during research presentations: If information from this study is published or presented at scientific meetings, and when your samples and information are shared with other researchers and deposited in data and cell/tissue storage banks, your name and other direct personal identifiers will not be used.

Use and sharing of your health information: We do not sell, rent, or lease your contact information.

We ask anyone who gets your information from us to protect the privacy of your information.

However, we cannot control how they may use or share your health information. We cannot promise that they will keep it completely private. When information or biological samples are shared with researchers beyond those listed in the section “What will happen in this research study?” your information is identified by a code and not with your name or other direct personal identifiers.

Third Party Involvement

For the purposes of the home visits with ExamOne staff, research staff will set up profiles for participants for scheduling and contact purposes, including name and contact details. This data, which is not stored at Boston Medical Center or Boston University, is outside our control.

If you do not want to share your contact information with ExamOne, you will not be able to have a home visit with ExamOne staff. You will still be a Framingham Heart Study participant.

Communicating with participants: This study gives you the option of communicating using unsecure email and/or text for appointment reminders and satisfaction surveys. This is because some people like the option of communicating by email and/or text message. It is important for you to understand that regular email and text are convenient but are generally not secure. As a result, information about you could be intercepted by someone not involved in the study. We will give you the option of using secure email (Data Motion), non-secure email, and/or non-secure text.

Certificate of Confidentiality (CoC): This study is covered by a Certificate of Confidentiality (CoC) from the National Institutes of Health. All studies funded by the National Institutes of Health that involve identifiable information or biological samples are covered by a CoC. The CoC specifies how we can share research information or biological samples. Because we have a CoC, we cannot give out research information or biological samples that may identify you to anyone that is not involved in the research except as we describe below. Even if someone tries to get your information or biological samples in connection with a legal proceeding, we cannot give it to them. The CoC does not prevent you from sharing your own research information.

If you agree to be in the study, we will share information and biological samples that may show your identity with the following groups of people:

- People who do the research or help oversee the research, including safety monitoring.
- People from Federal and state agencies who audit or review the research, as required by law. Such agencies may include the U.S. Department of Health and Human Services, the Food and Drug Administration, the National Institutes of Health, and the Massachusetts Department of Public Health.
- Researchers for studies described in the section "What will happen in this research study?" These people are expected to protect your information and biological samples in the same way we protect it.
- Any people who you give us separate permission to share your information.

We will share research data where we have removed anything that we think would show your identity. There still may be a small chance that someone could figure out that the information is about you. Such sharing includes:

- Publishing results in a medical book or journal.
- Adding results to a Federal government database.
- Using research data in future studies, done by us or by other scientists.
- Using biological samples in future studies, done by us or by other scientists.

Sharing of Genetic Data and Samples: Samples that are collected from you in this study will be analyzed to find out information about your genetic makeup. Your genetics and health information, without your name or other data that could easily identify you, will be put in a database run by the National Institutes of Health (NIH). This may include your whole genome information. Other researchers can ask the NIH to get your information from the database. You should know that it is possible that your genetic information might be used to identify you or your family, though we believe it is not likely that this will happen. Once your information is given to the NIH database, you can ask to have NIH stop sharing it, but NIH cannot take back information that was already shared.

You should know that we are required to report information about child abuse or neglect, elder abuse, specific reportable diseases, or harm to others.

How long will I be in the study?

FHS is a long-term study. Taking part in this research is up to you. You can decide to not take part. If you decide to take part now, you can change your mind and withdraw later.

We will tell you if we learn new information that could make you change your mind about taking part in this research study.

The investigator may decide to discontinue your participation without your permission because he/she may decide that staying in the study will be bad for you, or the sponsor may stop the study.

The Framingham Heart Study has always, and will always, seek your consent for future exam cycles. However, because FHS is a longitudinal study spanning many years, there may be a time when you cannot provide consent. In such a case, we would seek the necessary consent

to continue your participation from your current Research Proxy. If we are unable to contact your current Research Proxy, FHS may continue the core exams that you have already consented to under this Agreement. You or your Research Proxy can decline or discontinue participation at any point, and this will always be honored.

Subject's Rights

By consenting to be in this study you do not waive any of your legal rights. Consenting means that you have been given information about this study and that you agree to participate in the study. You will be given a copy of this form to keep.

If you do not agree to be in this study or if at any time you withdraw from this study you will not suffer any penalty or lose any benefits to which you are entitled. Your participation is completely up to you. Your decision will not affect your ability to get health care or payment for your health care. It will not affect your enrollment in any health plan or benefits you can get. You will only be paid for the study activities that you complete before withdrawing.

During this study, we may find out something that might make you not want to stay in the study. If this happens, we will tell you as soon as possible. You should also tell us if you ever have concerns about being in the study.

We may decide to have you stop being in the study even if you want to stay. Some reasons this could happen are if staying in the study may be bad for you, or if the study is stopped.

If I have questions or concerns about this research study, who can I call? (Questions)

The FHS research team will try to answer all of your questions. You can ask questions as often as you want. If you have questions or concerns at any time, contact a study staff member directly at (508) 872-6562 or (800) 854-7582. You can also send an email to FHS@bu.edu. Also call if you need to report an injury while being in this research study.

The Framingham Heart Study is led by investigators from Boston University and the National Heart, Lung, and Blood Institute (NHLBI) at the National Institutes of Health. Dr. Joanne Murabito and Dr. Donald Lloyd-Jones (Boston University), and Dr. Daniel Levy (NHLBI) are in charge of the research study.

- You can contact Drs. Murabito and Lloyd-Jones at (508) 872-6562 or (800) 854-7582 Monday through Friday between 9am and 5pm or by email at fhs@bu.edu.
- You may contact Dr. Levy at (508) 935-3400 Monday through Friday between 9am and 5pm or by email at levyd@nih.gov.

You may also call (617) 358-5372 or email medirb@bu.edu . You will be talking to someone at the Boston Medical Center and Boston University Medical Campus IRB. The IRB is a group that helps monitor research. You should call or email the IRB if you want to find out about your rights as a research subject. You should also call or email if you want to talk to someone who is not part of the study about your questions, concerns, or problems.

BMC and BU Medical Campus IRB
IRB NUMBER: H-32132
IRB APPROVAL DATE: 12/02/2025

Please read all of the following statements and make a selection for each item to indicate how FHS shares your data:

1. I agree to participate in the FHS examination, including the collection of data, blood, urine samples, and various research tests and measurements. I agree to the use of all data, samples and research materials for studies of the factors contributing to heart and blood vessel diseases, lung and blood diseases, stroke, memory loss, cancer, and other diseases and medical conditions.

   |___| YES |___| NO (for Internal Use- Office Code 0)

2. I agree to allow my data, blood, DNA and other genetic material, iPS cells and their derivatives, urine samples, and any other specimens to be used in genetic research of factors contributing to heart and blood vessel diseases, lung and blood diseases, stroke, memory loss, cancer, and other diseases and medical conditions.

   |___| YES |___| NO (for Internal Use- Office Code 3)

3. I agree to allow researchers from commercial companies to have access to my data, blood, DNA and other genetic material, iPS cells and their derivatives, urine samples, and any other specimens for research. I understand that my data and specimens will be shared without my name or direct personal identifiers.

   |___| YES |___| NO (for Internal Use- Office Code 4)

4. I agree to allow FHS to release the findings of non-genetic research tests and examinations to me and/or my physician, clinic, hospital, or other health care provider.

   |___| YES |___| NO (for Internal Use- Office Code 30)

5. I agree to allow FHS to provide me and, with my permission, my physician, clinic, hospital, or other health care provider relating to genetic research findings as they may relate to me.

   |___| YES |___| NO (for Internal Use- Office Code 31)

6. I agree to allow FHS to collect my Medicare and/or Medicaid Beneficiary Identification numbers for research purposes.

   |___| YES |___| NO (for Internal Use- Office Code 32)

BMC and BU Medical Campus IRB
IRB NUMBER: H-32132
IRB APPROVAL DATE: 12/02/2025

Subject:

____

Printed name of subject

By signing this consent form, you are indicating that:

- you have read this form (or it has been read to you)
- your questions have been answered to your satisfaction
- you voluntarily agree to participate in this research study
- you permit the use and sharing of information that may identify you as described including your health information

To be completed by subject if personally signing

______________________________________ ____________________

Signature of subject | Date

To be completed by LAR if subject does not personally sign

I am providing consent on behalf of the subject.

______________________________________ ____________________

Printed name of Legally Authorized Representative (LAR) | Relationship to Subject

______________________________________ ____________________

Signature of Legally Authorized Representative | Date

BMC and BU Medical Campus IRB
IRB NUMBER: H-32132
IRB APPROVAL DATE: 12/02/2025

Researcher:

____

Printed name of person conducting consent discussion

To be completed by researcher if subject personally signs

I have personally explained the research to the above-named subject and answered all questions. I believe the subject understands what is involved in the study and freely agrees to participate.

____________________________________ ____________________

Signature of person conducting consent discussion Date

To be completed by researcher if subject does not personally sign

I have personally explained the research to the above-named subject's Legally Authorized Representative and answered all questions. I believe that the Legally Authorized Representative understands what is involved in the study and freely agrees to have the subject participate.

I consider that the above-named subject (check one):

- Is capable of understanding what is involved in the study and freely agrees to participate.
- Is not capable of understanding what is involved in the study.

_____________________________ ___________________

Signature of Person conducting consent discussion Date

BMC and BU Medical Campus IRB
IRB NUMBER: H-32132
IRB APPROVAL DATE: 12/02/2025
