# Supplementary material for: Sex-Specific Fifteen-Year Alcohol Consumption Trajectories and Their Association with Cardiovascular Events and Mortality: The Framingham Heart Study
Source: Nutrients. 2026 Mar 5;18(5):849. doi: 10.3390/nu18050849 (PMC12986612; doi:10.3390/nu18050849)
Supplement: Supplementary file 1 [file nutrients-18-00849-s001.zip › H-41461_transomics_alcohol_updated.pdf]

BOSTON MEDICAL CENTER
EXCEPTIONAL CARE. WITHOUT EXCEPTION.

BOSTON UNIVERSITY

Institutional Review Board
72 E. Concord St., Robinson 4 – Suite 414
Boston, Massachusetts 02118-2307
Tel: 617-358-5372

Title of Study: Trans-omic Analysis of Alcohol Consumption and its Relation to Cardiovascular Disease
IRB Number: H-41461

RE: Continuing Review Submission Form
Review Type: Expedited
Action: Approved

Date of Action: March 21, 2024
Effective Date: 03/29/2024
Status Check-In Due Date: 03/28/2027

Funding Source: NIH/National Institute on Alcohol Abuse and Alcoholism (NIAAA)(NIH-NIAAA)
Award #: 1R01AA028263 - 01A1

March 21, 2024

Dear Chunyu Liu, PhD,

A qualified member of the Institutional Review Board (IRB) has reviewed the above referenced continuing review submission and determined that the study meets the requirements set forth by the IRB and is hereby approved for continuation. This submission was approved by the expedited review process in accordance with the policies and procedures of the Human Research Protection Program (http://www.bumc.bu.edu/ohra/hrpp-policies/hrpp-policies-procedures/#10.2.2).

No changes to the study have been approved as part of this continuing review.

Please note: this approval does NOT represent approval of any aspects of this study that have not been previously approved by the IRB.

This approval is valid through the expiration or status check-in due date indicated above.

This approval corresponds with the versions of the application and attachments in the electronic system most recently approved as of the date of this letter.

As a principal investigator, you are reminded that you must comply with the responsibilities listed here <http://www.bumc.bu.edu/irb/maintaining-irb-approval/responsibilities-of-the-principal-investigator/>.

Resources
The Clinical Research Resources Office (CRRO) has a number of tools, templated documents, and guidance available for the research community to use. These resources are highly recommended to help

teams meet protocol adherence and regulatory standards. Please review the available resources at the CRRO Resource Library.

Sincerely,

Lin Themelis, MA, CIP, IRB Administrator
