# Supplementary material for: Sex-Specific Fifteen-Year Alcohol Consumption Trajectories and Their Association with Cardiovascular Events and Mortality: The Framingham Heart Study
Source: Nutrients. 2026 Mar 5;18(5):849. doi: 10.3390/nu18050849 (PMC12986612; doi:10.3390/nu18050849)
Supplement: Supplementary file 1 [file nutrients-18-00849-s001.zip › Original-Cohort-Exam-29.pdf]

The Framingham Heart Study

Research Consent Form

Cohort Exam 29

Background
You are asked to participate in the 29th Framingham Heart Study examination. This is an observational research study designed to identify the relationship between risk factors, genetics, cardiovascular disease, and other health conditions.

Purpose
The purpose of this research study is to 1) investigate factors related to the development of heart and blood vessel diseases, lung and blood diseases, stroke, memory loss, cancer, and other diseases and health conditions; and 2) examine DNA and its relationship to the risks of developing these diseases and health conditions. This examination does not take the place of a routine physical examination by your physician.

What Happens In This Research Study
You will be one of approximately 440 subjects to be asked to participate in this study.

The research will take place at the following location(s): Boston University Medical Center.

Your research examination will take place at the Framingham Heart Study facility located at 73 Mt. Wayte Avenue in Framingham, MA. The examination will take approximately 2.5 hours and will include the following:

History An interview about your medical status including: heart and lung illnesses, hospitalizations, emergency room visits, day surgeries, physician visits, and medical health habits (including diet, prescription, and non-prescription drug use).

Measurements and Procedures You will be asked to participate in standardized measurements routinely done in your physician's office such as height, weight, blood pressure and electrocardiogram. The electrocardiogram measures the rate and regularity of your heartbeats. You will be asked questions to assess your ability to perform activities of daily living, general daily function, measures of memory and mood and questions about your social support. You will also be asked questions about your leisure time activities and health care preferences.

You will also be asked to perform tasks to assess your walk, balance, and hand grip strength. This is called Observed Physical Performance.

In the event that you may have had a stroke, you will be examined during your hospitalization (if applicable) and at 3, 6, 12, and 24 months. The examination will include a neurological evaluation and assessment of your ability to perform activities of daily living. If the neurologist believes that you have had a stroke or definite memory problems, you will be asked if you would be willing to have an M.R.I. (Magnetic Resonance Imaging) scan of the brain. If you do decide at that time to undergo the test, it will be arranged by the clinic coordinator. In some instances, you may be asked to return to the clinic for further testing based on information obtained from your examination.

Blood Specimen A technician will draw a sample of your blood (20 cc or about 1 Table-spoon) and use currently obtained or previously frozen samples (if applicable) of blood for testing of potential risk factors for the diseases and health conditions under investigation. The blood samples will also be tested for genetic studies.

Data and DNA will be distributed to Framingham Heart Study researchers and other qualified researchers interested in the genetics of heart and blood vessel diseases, lung and blood diseases, stroke, memory loss, joint disease, bone loss, deafness, cancer, and other major diseases and health conditions. The researchers will be given the DNA without any potentially identifying information. Information gained from research on your DNA may be used for the development of diagnostic procedures or new treatments for major diseases. Your DNA will not be sold to any person, institution, or company for financial gain or commercial profit. However, neither you nor your heirs will gain financially from discoveries made using the information and/or specimens that you provide.

Medical Records You will be asked to sign a medical release form to allow the Framingham Heart Study Medical Records staff to obtain and review copies of your hospital, cancer registry, and medical records for Framingham Heart Study physician review. This medical release form will be considered valid to obtain these records and this authorization will be valid until canceled by you.

With your permission, a summary letter of routine test results from this exam will be sent to you and your physician.

You will be contacted every 1-2 years to obtain additional health information. You may also be contacted to obtain determine your interest in participating in other FHS health-related studies. It is expected that this exam will be done approximately every 1-2 years at which time you will be asked to sign a new consent form. If an exam is not possible, you may be asked to complete a medical history update over the phone.

You may choose to withdraw your blood samples at a future date and your samples will be destroyed at that time. If you choose to withdraw your samples, you should call the Framingham Heart Study at (508) 935-3477 and ask for the lab manager.

The Framingham Heart Study is a medical research project sponsored by the National Institutes of Health. It is authorized under 42USC 285b-3. The system of records which applies to the Framingham Heart Study is documented in the Federal Register: September 26, 2002 (Vol. 67, No. 1879) pages 60776-60780.

Risks and Discomforts
Each of the test procedures and their risks and discomforts are listed below:

The Blood Draw Minimal bruising, pain, or bleeding may occur as a result of the blood draw. A latex allergy can occur from the gloves worn by the technician. If you have a known latex allergy, inform the technician and he/she will use another form of protection.

Observed Physical Performance This test involves a very low level of risk. The primary risk involved is injury from falling.

We do not expect an unusual risk or injury to occur as a result of participation. In the unlikely event that during examination procedures you should require medical care, first aid will be available.

There may be unknown risks/discomforts involved. Study staff will update you in a timely way on any new information that may affect your health, welfare, or decision to stay in this study

Potential Benefits
You will receive no direct benefit from your participation in this study. However, your participation may help the investigators better understand the cause and prevention of cardiovascular disease and other health conditions, including the possibility of how genetic factors influence health status.

Alternatives
There are no alternative treatments or procedures to participating in this study. You may, however, choose not to participate.

Subject Costs and Payments
You will not be charged for any part of the examination. If the examination uncovers any medical problems that require medical diagnosis or treatment, you will be so advised and that information will be provided to the physician or clinic that you choose.

In the event that your physician decides that follow up clinical tests or treatments are necessary, payment must be provided by you or your third party payer, if applicable (for example, health insurance or Medicare). No special arrangements will be made by the Framingham Heart Study for compensation or for payment of treatment solely because of your participation in this study. This does not waive any of your legal rights.

Costs that you might incur the day of your participation include, but are not limited to, transportation costs (gas, tolls, etc). You will not receive payment for your participation. However, if necessary, we will provide transportation to the clinic and your return home at no cost.

Confidentiality
Any information we obtain about you during this study will be treated as strictly confidential to the full extent permitted by applicable law. To ensure confidentiality, a code number will be assigned to you and any of your potentially identifying information.

The code number will not be used on any blood samples you provide. A label with a new security bar code number and the date the specimen is drawn will be the only information on the label. The code numbers will only be provided to qualified investigators studying the DNA samples. Files linking names to samples will be kept locked and accessible only to Framingham Heart Study data managers. The coded samples will be stored securely, separated from files which link your name to the code numbers.

No other individuals will have access to the stored sample or information gained from your stored sample. Because no information will be provided to you or to others from the analysis of this sample, the risk in providing this sample is minimal. Your sample will be kept until it is no longer of scientific value.

When study results are published, your name and any other potentially identifying information (i.e. code number) will not be revealed. You will be kept informed through periodic publications from the Framingham Heart Study of any new findings about genetics, cardiovascular disease or other health conditions generated from the DNA analyses.

Information from this study and from your medical record may be reviewed and photocopied by the Food and Drug Administration (FDA) and/or state and federal regulatory agencies such as the Office of Human Research Protection as applicable, and the Institutional Review Board of Boston University Medical Center.

Please check the appropriate box beside each statement:

1) |___| YES |___| NO I agree to participate in the Framingham Heart Study examination described above to study the frequency of and factors contributing to heart and blood vessel diseases, lung and blood diseases, stroke, memory loss and other diseases and health conditions.

2) |___| YES |___| NO I agree to provide a blood sample from which DNA and other components can be extracted. The DNA will be made available to researchers studying the diseases listed above.

3) |___| YES |___| NO I agree to allow researchers from private companies to have access to my DNA and genetic data which may be used to develop diagnostic lab tests or pharmaceutical therapies that could benefit many people. (Note: You or your heirs will not benefit financially from this, nor will your DNA be sold to anyone.)

4) |___| YES |___| NO I agree to allow the Framingham Heart Study to release the findings from non-genetic tests and examinations to my physician, clinic, or hospital.

5) |___| YES |___| NO If a genetic condition is identified that may have potentially important health and treatment implications for me, I agree to allow the Framingham Heart Study to notify me, and with my permission, to notify my physician.

Subject's Rights

By signing this form you do not waive any of your legal rights. Signing this consent form means that you have heard or read the information about this study and that you agree to participate. You will be given a copy of this signed form to keep.

You may obtain further information about your rights as a research subject by calling the Office of the Institutional Review Board of Boston University Medical Center at 617-638-7207.

The investigator and/or his/her designee will try to answer all of your questions. If you have questions or concerns at any time, or if you need to report an injury related to the research, contact PHILIP WOLF at (508) 872-6562.

Compensation for Research Related Injury

If you think that you have been injured by being in this study, please let the investigator know right away. If your part in this study takes place at Boston Medical Center, you can get treatment for the injury at Boston Medical Center. If your part in the study is not at Boston Medical Center, ask the investigator where treatment for injury would be available locally. You and your insurance company will be billed for this treatment. Some research sponsors may offer a program to cover some of the treatment costs which are not covered by your insurance. You should ask the research team if such a program is available.

Right to Refuse or Withdraw
Taking part in this study is voluntary. You have the right to refuse to take part in this study. If you decide to be in the study and then change your mind, you can withdraw from the research. Your participation is completely up to you. Your decision will not affect your being able to get health care at this institution or payment for your health care. It will not affect your enrollment in any health plan or benefits you can get. If you choose to take part, you have the right to stop at any time. If there are any new findings during the study that may effect whether you want to continue to take part, you will be told about them as soon as possible. The investigator may decide to discontinue your participation without your permission because he/she may decide that staying in the study will be bad for you, or the sponsor may stop the study.

Signing this consent form indicates that you have read this consent form (or have had it read to you), that your questions have been answered to your satisfaction, and that you voluntarily agree to participate in this research study. You will receive a copy of this signed consent form.

Subject

____ ____________________

Signature Date

____

Printed Name

Person Obtaining Consent

____ ____________________

Signature Date

____

Printed Name

Cohort - Exam 29
Res.v8
