# Supplementary material for: Sex-Specific Fifteen-Year Alcohol Consumption Trajectories and Their Association with Cardiovascular Events and Mortality: The Framingham Heart Study
Source: Nutrients. 2026 Mar 5;18(5):849. doi: 10.3390/nu18050849 (PMC12986612; doi:10.3390/nu18050849)
Supplement: Supplementary file 1 [file nutrients-18-00849-s001.zip › revised_Supplemental_022426.pdf]

SUPPLEMENTAL MATERIALS

Fifteen-year Alcohol Consumption Trajectories and Their Association with Cardiovascular Events and Mortality: the Framingham Heart Study

SUPPLEMENTAL METHODS

Collection of alcohol intake information using the FFQ questionnaire

A stand-alone questionnaire was used to collect the information on alcohol consumption during each health examination. This questionnaire is designed to gather information on the typical frequency and types of alcoholic beverages, including beer, wine, or liquor, that participants consumed on average per week over the past year. For each type of beverage, the questionnaire inquired about the participant's consumption by asking: 1) the number of days they drank, and 2) the average number of drinks consumed. A serving is defined as one bottle or drink of beer (12 oz.), a glass of white or red wine or rosé (5 oz.), or a glass of liquor (such as a cocktail or highball) (1.5 oz.). The continuous phenotype, "grams of alcohol consumed per day", was calculated using the following converters between drinks and grams: one beer (12 oz.), one glass of red or white wine (5 oz.) or one drink of spirit (1.5 oz. 80 proof alcohol) was approximately 14 grams ethanol.

Sensitivity analysis: covariates definition

PAI was calculated as a composite score based on self-reported sleeping hours and engaging in sedentary, slight, moderate, and heavy activities over 24 hours. Weights of 1, 1.1, 1.5, 2.4, and 5 were assigned to sleep, sedentary, slight, moderate, and heavy activity, respectively [1, 2]. Marital status was grouped into married or unmarried. Occupation was classified into eight categories: professional, executive, supervisory, technical, laborer, clerical, sales, and housewife. Occupation was used as a proxy for income, which was not collected in the respective FHS exams. Cancer data was gathered based on histological or morphological cell histopathology, coded as a binary variable indicating whether a participant had cancer at the baseline of Phase 2.

Sensitivity analysis: statistical model description

In Phase 2, we conducted four sensitivity analyses: (1) We assessed whether combining similar alcohol consumption trajectory groups affected the association results, specifically by testing whether five groups could be reduced to four and using the combined group used as the reference. (2) We also compared models that included ASRS and FRS as covariates, instead of individual CVD risk factors, to our main model. (3) We evaluated additional potential confounders, including physical activity index (PAI), marital status, occupation category, and cancer status. However, due to missing data, including all of these variables in the main model would have substantially reduced the sample size and potentially introduced bias. Descriptions of these variables are in the Supplemental Materials. We matched these variables contemporaneously with or as close as possible to the baseline of the Phase 2 study. (4) We included family structure as a random effect to account for parent-offspring correlations and to reduce confounding from shared genetic and environmental influences, as many participants in the Original and Offspring cohorts belong to the same families.

SUPPLEMENTAL RESULTS

Secondary analyses of associations between alcohol trajectories and all-cause mortality

In secondary analyses, we repeated sex-specific models separately in the Original and Offspring cohorts to assess cohort-specific consistency (**Supplemental Materials**, **Supplemental Table 9**). Among women, significant associations were observed only in the Original cohort. In the Original cohort, compared to the reference group, the Low-to-None drinking group had a 37% higher risk of mortality (95% CI = 1.12-1.67, $P = 0.002$), the Inverse-U Pattern drinking group had a 34% higher risk (95% CI = 1.07-1.68, $P = 0.010$), and High-Decreasing drinking group had a 34% higher risk (95% CI = 1.05-1.70, $P = 0.017$) after adjusting for the same set of

covariates at Phase 2 baseline. In the Offspring cohort, mortality risks in the Low-to-None and High-Decreasing drinking groups were similar to the reference group. The Inverse-U Pattern drinking group showed a 7% higher risk, but the association was not statistically significant, adjusting for the same covariates (**Supplemental Table 9**). Among men, all associations displayed the same directions between the Original cohort and Offspring cohort. However, in the Original cohort, the High-Decreasing drinking group had a 40% increased risk of mortality compared to the reference group (95% CI = 1.14-1.72, $P = 0.001$), but this association was not observed in the Offspring cohort (**Supplementary Table 9**).

Secondary analyses of associations between alcohol trajectories and CHD

In both men and women, cohort-specific analyses demonstrated consistent directions of association when comparing CHD risk across trajectory groups relative to the reference group, supporting the rationale for the combined cohort analysis. Among women, the other three groups generally exhibited higher CHD risk compared to the reference group; however, only the Inverse-U Pattern drinking group in the Original cohort (HR = 1.90, 95% CI: 1.22–2.97, $P = 0.005$) and the Low-to-None drinking group in the Offspring cohort (HR = 1.90, 95% CI: 1.07–3.36, $P = 0.03$) showed statistically significant associations. Among men, the Low-to-None and high-decreasing drinking groups showed consistent associations with CHD risk compared to the reference group, with significantly higher risk observed for the Low-to-None drinking group in the Offspring cohort (HR = 2.03, 95% CI: 1.39–2.95, $P < 0.001$) and high-decreasing drinking group in the Offspring cohort (HR = 1.83, 95% CI: 1.12–3.00, $P = 0.017$) (**Supplemental Table 9**).

Summary of secondary analyses

Results were broadly consistent across cohorts within each sex. The combined analysis, which integrated both cohorts, produced intermediate effect estimates but demonstrated stronger statistical significance and narrower confidence intervals, indicating improved precision and stability of the associations.

Sensitivity analysis to evaluate additional variables

We conducted several sensitivity analyses to evaluate the choice of reference group and to assess the robustness of associations between alcohol trajectory groups and outcomes under different covariate specifications. First, the risks of all-cause mortality and incident CHD were similar when comparing the two Moderate-Decreasing drinking groups, Moderate-Decreasing drinking group A alone versus the combined Moderate-Decreasing drinking groups A and B as the reference (Supplemental Table 8). Second, results remained consistent when substituting individual CVD risk factors with composite scores such as ASRS or FRS as covariates (Supplemental Figures 6–7). Third, associations were largely unchanged in models that included additional covariates, including physical activity index (PAI), marital status, occupation, and cancer status, compared to models without them, using the same sample size (Supplemental Figures 8). These variables were excluded from the primary models due to missing values, which could greatly reduce sample size and introduce bias. Finally, the results remain largely unchanged after adjusting for family structure (Supplemental Figure 9).

SUPPLEMENTAL TABLES

Supplemental Table S1. Characteristics of the study participants at baseline of Phase 2

Variable[1]	Men (n=2935)	Women (n=3635)	*P* value
Age, years	65 (12)	67 (13)	< 0.001
BMI, kg/m[2]	27.7 (4.2)	26.7 (5.2)	< 0.001
DBP, mmHg	80.6 (11.7)	77.7 (11.8)	< 0.001
SBP, mmHg	140.3 (22.8)	141.0 (26.7)	0.277
TC, mg/dL	201.3 (40.4)	216.1 (40.7)	< 0.001
HDL, mg/dL[2]	42.8 (12.5)	55.6 (16.1)	< 0.001
FRS	27.3 (19.4)	14.0 (13.8)	< 0.001
ASRS	19.3 (15.2)	12.0 (12.6)	< 0.001
Education, *n* (%)			< 0.001
No high school	564 (19.2%)	721 (19.8%)	
High school	865 (29.5%)	1225 (33.7%)	
Some college	589 (20.1%)	988 (27.2%)	
College or above	917 (31.2%)	701 (19.3%)	
Hypertension treatment, *n* (%)	1021 (35.0%)	1311 (36.2%)	0.345
Lipid treatment, *n* (%)	298 (10.2%)	241 (6.6%)	< 0.001
Current diabetes, *n* (%)	361 (12.6%)	291 (8.4%)	< 0.001
Obesity, *n* (%)	699 (24.6%)	738 (21.9%)	0.011
Hypertension[3], *n* (%)	1588 (54.1%)	1969 (54.2%)	0.95
Alcohol consumption, g/day[4]	6 (0, 22)	0 (0, 8)	< 0.001
Incident CVD, *n* (%)	445 (15.2%)	493 (13.6%)	0.065
Incident CHD, *n* (%)	312 (10.6%)	263 (7.2%)	< 0.001
All-cause mortality, *n* (%)	1141 (38.9%)	1124 (30.9%)	< 0.001
Mortality follow up, years[4]	10 (4, 10)	10 (7, 10)	< 0.001
CVD follow up, years[4]	10 (5, 10)	10 (7, 10)	< 0.001
CHD follow up, years[4]	10 (5, 10)	10 (7, 10)	< 0.001

[1]Values were presented as mean (SD) for continuous variables and count (percent) for category variables.
[2]HDL information was not available for Original cohort at exam 12.
[3]Hypertension was defined as the use of hypertension treatment, SBP ≥130 mmHg, or diastolic blood pressure (DBP) ≥80 mmHg.
[4]Median (interquartile range [IQR]) was reported.
BMI, body mass index; DBP/SBP, diastolic/systolic blood pressure; TC, total cholesterol; HDL, high-density lipoprotein; FRS, the Framingham risk score; ASRS, atherosclerotic cardiovascular disease risk score; CVD, cardiovascular disease; CHD, coronary heart disease.

Supplemental Table S2. Women: Characteristics of the study sample at baseline of Phase 2, stratified by alcohol consumption trajectory groups

Variable[1]	Trajectory group[5]				
	Moderate-Decreasing (n=1179)	Low-to-None (n=992)	Inverse-U Pattern (n=606)	High-Decreasing (n=858)	*P* value
Age, years	66 (13)	69 (13)	69 (12)	63 (12)	< 0.001
BMI, kg/m^2	27.0 (5.1)	27.7 (5.6)	26.7 (5.2)	25.5 (4.5)	< 0.001
DBP, mmHg	77.2 (11.7)	77.8 (12.2)	78.4 (11.9)	77.6 (11.5)	0.284
SBP, mmHg	139.5 (27.0)	143.9 (26.3)	143.8 (27.4)	137.6 (25.6)	< 0.001
TC, mg/dL	216.4 (40.5)	214.2 (40.5)	216.5 (41.6)	217.3 (40.4)	0.493
HDL, mg/dL[2]	54.9 (15.0)	50.3 (13.7)	54.0 (15.3)	62.5 (17.8)	< 0.001
FRS	13.5 (14.3)	15.5 (14.2)	15.9 (14.7)	12.3 (12.3)	< 0.001
ASRS	11.4 (12.6)	13.4 (13.1)	14.4 (13.7)	10.0 (11.1)	< 0.001
Education, *n* (%)					< 0.001
No high school	216 (18.3%)	280 (28.2%)	137 (22.6%)	88 (10.3%)	
High school	404 (34.3%)	356 (35.9%)	204 (33.7%)	261 (30.4%)	
Some college	330 (28.0%)	244 (24.6%)	166 (27.4%)	248 (28.9%)	
College or above	229 (19.4%)	112 (11.3%)	99 (16.3%)	261 (30.4%)	
Hypertension treatment, *n* (%)	401 (34.2%)	413 (41.8%)	245 (40.5%)	252 (29.4%)	< 0.001
Lipid treatment, *n* (%)	89 (7.6%)	71 (7.2%)	36 (5.9%)	45 (5.2%)	0.156
Current diabetes, *n* (%)	101 (8.9%)	113 (12.4%)	42 (7.4%)	35 (4.2%)	< 0.001
Obesity, *n* (%)	244 (22.1%)	248 (28.6%)	121 (21.6%)	125 (14.9%)	< 0.001
Hypertension[3], *n* (%)	606 (51.4%)	602 (60.7%)	358 (59.1%)	403 (47.0%)	< 0.001
Alcohol consumption, g/day[4]	2 (0, 4)	0 (0, 0)	0 (0, 2)	16 (10, 38)	< 0.001
Incident CVD, *n* (%)	132 (11.2%)	105 (10.6%)	61 (10.1%)	112 (13.1%)	0.253
Incident CHD, *n* (%)	50 (4.2%)	44 (4.4%)	27 (4.5%)	45 (5.2%)	0.741
All-cause mortality, *n* (%)	372 (31.6%)	307 (30.9%)	227 (37.5%)	293 (34.1%)	0.030
Mortality follow up, years[4]	10 (9, 10)	10 (4, 10)	10 (6, 10)	10 (10, 10)	< 0.001
CVD follow up, years[4]	10 (8, 10)	10 (5, 10)	10 (5, 10)	10 (9, 10)	< 0.001
CHD follow up, years[4]	10 (10, 10)	10 (5, 10)	10 (5, 10)	10 (10, 10)	< 0.001

Analysis of covariance (ANCOVA) was used to compare mean differences across the trajectory groups.
[1]Values were presented as mean (SD) for continuous variables and count (percent) for category variables.
[2]HDL information was not available for the Original cohort at exam 12.
[3]Hypertension was defined as the use of hypertension treatment, SBP ≥130 mmHg, or diastolic blood pressure (DBP) ≥80 mmHg.
[4]Median (interquartile range [IQR]) was reported.
BMI, body mass index; DBP/SBP, diastolic/systolic blood pressure; TC, total cholesterol; HDL, high-density lipoprotein; FRS, the Framingham risk score; ASRS, atherosclerotic cardiovascular disease risk score; CVD, cardiovascular disease; CHD, coronary heart disease.
[5] The Moderate-Decreasing drinking group consisted of women with moderate alcohol intake (0-14 g/day) showing a decreasing trend. The Low-to-None drinking group primarily included long-term abstainers. The Inverse-U Pattern drinking group comprised women with varying alcohol intake patterns, while the High-Decreasing drinking group included women with consistently high intake levels (>14 g/day), also showing a decreasing trend.

Supplemental Table S3. Women: Association between alcohol consumption trajectories and all-cause mortality during a 10-year follow-up period

Model	Trajectory group[2]	Beta	HR[3]	95% CI[3]	*P* value
Multi-adjusted[1]	Moderate-Decreasing			Reference	
	Low-to-None	0.22	1.25	(1.05, 1.49)	**0.0134**
	Inverse-U Pattern	0.25	1.28	(1.05, 1.56)	**0.0136**
	High-Decreasing	0.21	1.24	(1.01, 1.52)	**0.0400**
Unadjusted	Moderate-Decreasing			Reference	
	Low-to-None	0.21	1.24	(1.04, 1.48)	**0.0176**
	Inverse-U Pattern	0.22	1.24	(1.02, 1.51)	**0.0305**
	High-Decreasing	0.20	1.22	(1.00, 1.48)	0.0533

Cox proportional hazards models were used for all analyses to examine the association between alcohol consumption trajectory groups and time to all-cause mortality.

[1] Covariates included age, education level, BMI, current smoking status, SBP, hypertension treatment, and diabetes at the baseline of Phase 2.

[2] The Moderate-Decreasing drinking group consisted of women with moderate alcohol intake (0-14 g/day) showing a decreasing trend. The Low-to-None drinking group primarily included long-term abstainers. The Inverse-U Pattern drinking group comprised women with varying alcohol intake patterns, while the High-Decreasing drinking group included women with consistently high intake levels (>14 g/day), also showing a decreasing trend.

[3] HR, hazard ratio. 95% CI, 95% confidence interval.

Supplemental Table S4. Men: Characteristics of the study sample at baseline of Phase 2 stratified by alcohol consumption trajectory groups

Variable[1]	Trajectory group				
	Moderate-Decreasing (n=1534)	Low-to-None (n=826)	Inverse-U Pattern (n=199)	High-Decreasing (n=376)	*P* value
Age, years	65 (12.10)	64 (12)	65 (12)	66 (11)	0.006
BMI, kg/m^2	27.5 (4.1)	28.0 (4.5)	27.8 (4.1)	27.4 (3.9)	0.033
DBP, mmHg	80.6 (11.9)	79.6 (11.1)	79.1 (10.9)	83.1 (11.8)	< 0.001
SBP, mmHg	140.7 (23.9)	137.8 (21.2)	137.1 (20.6)	145.8 (22.1)	< 0.001
TC, mg/dL	202.6 (37.3)	196.7 (39.6)	195.4 (34.8)	209.3 (53.5)	< 0.001
HDL, mg/dL[2]	43.6 (12.5)	39.0 (10.9)	42.3 (11.5)	48.7 (13.9)	< 0.001
FRS	26.2 (18.9)	28.2 (20.0)	27.1 (18.6)	30.1 (20.2)	0.018
ASRS	19.0 (15.0)	19.2 (15.7)	20.1 (15.4)	20.7 (15.0)	0.403
Education, *n* (%)					0.212
No high school	287 (18.7%)	160 (19.4%)	36 (18.1%)	81 (21.5%)	
High school	423 (27.6%)	265 (32.1%)	61 (30.7%)	116 (30.9%)	
Some college	313 (20.4%)	165 (20.0%)	45 (22.6%)	66 (17.6%)	
College or above	511 (33.3%)	236 (28.6%)	57 (28.6%)	113 (30.1%)	
Lipid treatment, *n* (%)	144 (9.4%)	96 (11.6%)	19 (9.6%)	39 (10.4%)	0.403
Current diabetes, *n* (%)	149 (10.0%)	146 (18.0%)	27 (14.4%)	39 (10.6%)	< 0.001
Obesity, *n* (%)	348 (23.5%)	214 (26.8%)	47 (25.8%)	90 (24.3%)	0.351
Hypertension[3], *n* (%)	815 (53.1%)	435 (52.7%)	99 (49.7%)	239 (63.6%)	< 0.001
Alcohol consumption, g/day[4]	12 (4, 20)	0 (0, 0)	0 (0, 2)	44 (34, 64)	< 0.001
Incident CVD, *n* (%)	132 (8.6%)	72 (8.7%)	22 (11.1%)	38 (10.1%)	0.580
Incident CHD, *n* (%)	57 (3.7%)	36 (4.4%)	11 (5.5%)	17 (4.5%)	0.593
All-cause mortality, *n* (%)	436 (28.4%)	201 (24.3%)	62 (31.2%)	114 (30.3%)	0.056
Mortality follow up, years[4]	10 (4, 10)	10 (5, 10)	10 (6, 10)	10 (3, 10)	0.080
CVD follow up, years[4]	10 (5, 10)	10 (5, 10)	10 (6, 10)	10 (3, 10)	0.024
CHD follow up, years[4]	10 (5, 10)	10 (5, 10)	10 (5, 10)	10 (3, 10)	0.022

Analysis of covariance (ANCOVA) was used to compare mean differences across the trajectory groups.
[1]Values were presented as mean (SD) for continuous variables and count (percent) for category variables.
[2]HDL information was not available for the Original cohort at exam 12.
[3]Hypertension was defined as the use of hypertension treatment, SBP ≥130 mmHg, or diastolic blood pressure (DBP) ≥80 mmHg.
[4]Median (interquartile range [IQR]) was reported.
BMI, body mass index; DBP/SBP, diastolic/systolic blood pressure; TC, total cholesterol; HDL, high-density lipoprotein; FRS, the Framingham risk score; ASRS, atherosclerotic cardiovascular disease risk score; CVD, cardiovascular disease; CHD, coronary heart disease.
The Moderate-Decreasing drinking group consisted of men with moderate alcohol intake (0-28 g/day) showing a decreasing trend. The Low-to-None drinking group primarily included long-term abstainers. The Inverse-U Pattern drinking group comprised men with varying alcohol intake patterns, while the High-Decreasing drinking group included men with consistently high intake levels (>40 g/day), also showing a decreasing trend.

Supplemental Table S5. Men: Longitudinal association between alcohol consumption trajectories and all-cause mortality during a 10-year follow-up period

Model	Trajectory group[2]	Beta	HR[3]	95% CI[3]	*P* value
Multi-adjusted[1]	Moderate-Decreasing			Reference	
	Low-to-None	0.16	1.17	(1.01, 1.36)	**0.0357**
	Inverse-U Pattern	-0.16	0.85	(0.64, 1.12)	0.2450
	High-Decreasing	0.24	1.27	(1.07, 1.52)	**0.0073**
Unadjusted	Moderate-Decreasing			Reference	
	Low-to-None	0.21	1.23	(1.06, 1.42)	**0.0054**
	Inverse-U Pattern	-0.11	0.90	(0.68, 1.18)	0.4467
	High-Decreasing	0.27	1.31	(1.10, 1.56)	**0.0029**

Cox proportional hazards models were used for all analyses to examine the association between alcohol consumption trajectory groups and time to all-cause mortality.

[1] Covariates included age, education level, BMI, current smoking status, SBP, hypertension treatment, and diabetes at the baseline of Phase 2.

[2]The Moderate-Decreasing drinking group consisted of women with moderate alcohol intake (0-24 g/day) showing a decreasing trend. The Low-to-None drinking group primarily included long-term abstainers. The Inverse-U Pattern drinking group comprised women with varying alcohol intake patterns, while the High-Decreasing drinking group included women with consistently high intake levels (>40 g/day), also showing a decreasing trend.

[3]HR, hazard ratio. 95% CI, 95% confidence interval.

Supplemental Table S6. Women: Longitudinal association between alcohol consumption trajectories and incident CHD during a 10-year follow-up period

Model	Trajectory group[2]	Beta	HR[3]	95% CI[3]	*P* value
Multi-adjusted[1]	Moderate-Decreasing			Reference	
	Low-to-None	0.46	1.58	(1.12, 2.24)	**0.0090**
	Inverse-U Pattern	0.45	1.58	(1.08, 2.3)	**0.0183**
	High-Decreasing	0.48	1.61	(1.10, 2.35)	**0.0140**
Unadjusted	Moderate-Decreasing			Reference	
	Low-to-None	0.45	1.57	(1.11, 2.21)	**0.0101**
	Inverse-U Pattern	0.43	1.53	(1.05, 2.23)	**0.0262**
	High-Decreasing	0.39	1.47	(1.02, 2.13)	**0.0403**

Cox proportional hazards models were used for all analyses to examine the association between alcohol consumption trajectory groups and time to all-cause mortality.

[1]Covariates included age, education level, BMI, current smoking status, SBP, hypertension treatment, and diabetes at the baseline of Phase 2.

[2]The Moderate-Decreasing drinking group consisted of men with moderate alcohol intake (0-28 g/day) showing a decreasing trend. The Low-to-None drinking group primarily included long-term abstainers. The Inverse-U Pattern drinking group comprised women with varying alcohol intake patterns, while the High-Decreasing drinking group included men with consistently high intake levels (>40 g/day), also showing a decreasing trend (see Figure 3).

[3]HR, hazard ratio. 95% CI, 95% confidence interval.

Supplemental Table S7. Men: Longitudinal association between alcohol consumption trajectories and coronary heart disease (CHD) during a 10-year follow-up period

Model	Trajectory group[2]	Beta	HR[3]	95% CI[3]	*P* value
Multi-adjusted[1]	Moderate-Decreasing			Reference	
	Low-to-None	0.47	1.60	(1.23, 2.09)	**0.0005**
	Inverse-U Pattern	0.08	1.09	(0.69, 1.73)	0.7198
	High-Decreasing	0.23	1.26	(0.90, 1.77)	0.1818
Unadjusted	Moderate-Decreasing			Reference	
	Low-to-None	0.47	1.60	(1.23, 2.08)	**0.0005**
	Inverse-U Pattern	0.15	1.16	(0.73, 1.83)	0.5289
	High-Decreasing	0.28	1.33	(0.95, 1.85)	0.1001

Cox proportional hazards models were used for all analyses to examine the association between alcohol consumption trajectory groups and time to CHD.

[1]Covariates included age, education level, BMI, current smoking status, SBP, hypertension treatment, and diabetes at the baseline of Phase 2.

[2]The Moderate-Decreasing drinking group consisted of men with moderate alcohol intake (0-28 g/day) showing a decreasing trend. The Low-to-None drinking group primarily included long-term abstainers. The Inverse-U Pattern drinking group comprised women with varying alcohol intake patterns, while the High-Decreasing drinking group included men with consistently high intake levels (>40 g/day), also showing a decreasing trend (see Figure 3).

[3]HR, hazard ratio; 95% CI, 95% confidence interval

Supplementary Table S8. The Sex-specific association analysis between alcohol consumption trajectory groups with all-cause mortality and coronary heart disease (CHD) for participants in the Original cohort, the offspring cohort, and the combined sample during a 10-year follow-up period

			All-cause Mortality			Coronary Heart Disease		
Sex	Cohort	Trajectory Groups[2]	HR (95% CI)[1]	P value	n (cases)	HR (95% CI)[1]	P value	n (cases)
Women	Combined	Moderate-Decreasing	Reference		1078 (238)	Reference		958 (57)
		Low-to-None	1.25 (1.05, 1.49)	**0.0134**	836 (263)	1.58 (1.12, 2.24)	**0.009**	717 (76)
		Inverse-U Pattern	1.28 (1.05, 1.56)	**0.0136**	540 (171)	1.58 (1.08, 2.30)	**0.0183**	474 (52)
		High-Decreasing	1.24 (1.01, 1.52)	**0.04**	824 (169)	1.61 (1.10, 2.35)	**0.014**	770 (56)
	Original	Moderate-Decreasing	Reference		395 (176)	Reference		305 (36)
		Low-to-None	1.37 (1.12, 1.67)	**0.0024**	375 (212)	1.50 (0.97, 2.32)	0.0712	283 (47)
		Inverse-U Pattern	1.34 (1.07, 1.68)	**0.0102**	303 (142)	1.90 (1.22, 2.97)	**0.0047**	248 (44)
		High-Decreasing	1.34 (1.05, 1.70)	**0.017**	276 (123)	1.50 (0.91, 2.49)	0.1121	241 (29)
	Offspring	Moderate-Decreasing	Reference		683 (62)	Reference		653 (21)
		Low-to-None	0.96 (0.66, 1.40)	0.8201	461 (51)	1.90 (1.07, 3.36)	**0.0281**	434 (29)
		Inverse-U Pattern	1.07 (0.68, 1.68)	0.7612	237 (29)	0.99 (0.44, 2.25)	0.984	226 (8)
		High-Decreasing	1.01 (0.68, 1.50)	0.9555	548 (46)	1.74 (0.97, 3.12)	0.0621	529 (27)
Men	Combined	Moderate-Decreasing	Reference		1461 (534)	Reference		1161 (141)
		Low-to-None	1.17 (1.01, 1.36)	**0.0357**	792 (280)	1.60 (1.23, 2.09)	**0.0005**	611 (93)
		Inverse-U Pattern	0.85 (0.64, 1.12)	0.245	179 (56)	1.09 (0.69, 1.73)	0.7198	150 (21)
		High-Decreasing	1.27 (1.07, 1.52)	**0.0073**	366 (162)	1.26 (0.90, 1.77)	0.1818	293 (45)
	Original	Moderate-Decreasing	Reference		613 (394)	Reference		418 (93)
		Low-to-None	1.17 (0.96, 1.42)	0.1141	180 (142)	1.15 (0.73, 1.81)	0.5434	105 (25)
		Inverse-U Pattern	0.93 (0.66, 1.32)	0.6881	53 (35)	0.63 (0.28, 1.45)	0.2774	39 (6)
		High-Decreasing	1.40 (1.14, 1.72)	**0.0015**	164 (121)	0.99 (0.61, 1.60)	0.9613	115 (21)
	Offspring	Moderate-Decreasing	Reference		848 (140)	Reference		743 (48)
		Low-to-None	1.17 (0.92, 1.49)	0.21	612 (138)	2.03 (1.39, 2.95)	**0.0002**	506 (68)
		Inverse-U Pattern	0.77 (0.49, 1.22)	0.2705	126 (21)	1.66 (0.92, 2.99)	0.0909	111 (15)
		High-Decreasing	1.04 (0.73, 1.47)	0.8381	202 (41)	1.83 (1.12, 3.00)	**0.0167**	178 (24)

Cox proportional hazards models were used for all analyses to examine the association between alcohol consumption trajectory groups and time to all-cause mortality and CHD. Covariates included age, education level, BMI, current smoking status, SBP, hypertension treatment, and diabetes at the baseline of Phase 2.
[1]HR, hazard ratio; 95% CI, 95% confidence interval.
[2]The alcohol consumption groups were described in Figure 3. The Moderate-Decreasing drinking group included moderate drinkers (<14 g/day for women and <28 g/day for men) who slightly decreased their consumption. The Inverse-U Pattern drinking group comprised participants with varying alcohol intake patterns, while the High-Decreasing drinking group included participants with consistently high intake levels (>28 g/day for women and >40 g/day for men), also showing a decreasing trend.

Supplementary Table S9. The Sex-stratified association analysis between 5 unmerged alcohol consumption trajectory groups with CHD and all-cause mortality during a 10-year follow-up period

		All-cause Mortality			Coronary Heart Disease		
Sex	Trajectory group[2]	HR (95%)[1]	P value	n (cases)	HR (95%)[1]	P value	n (cases)
Women	Moderate-Decreasing: A	Reference		632 (127)	Reference		558 (27)
	Moderate-Decreasing: B	1.13 (0.87, 1.45)	0.3668	446 (111)	1.31 (0.78, 2.20)	0.3138	400 (30)
	Low-to-None	1.32 (1.06, 1.63)	**0.0111**	836 (263)	1.81 (1.16, 2.81)	**0.0085**	717 (76)
	Inverse-U Pattern	1.35 (1.07, 1.70)	**0.0103**	540 (171)	1.80 (1.13, 2.87)	**0.0138**	474 (52)
	High-Decreasing	1.31 (1.03, 1.65)	**0.0268**	824 (169)	1.84 (1.15, 2.93)	**0.0109**	770 (56)
Men	Moderate-Decreasing: A	Reference		724 (282)	Reference		556 (64)
	Moderate-Decreasing: B	0.88 (0.75, 1.05)	0.158	737 (252)	1.10 (0.79, 1.53)	0.5822	605 (77)
	Low-to-None	1.11 (0.93, 1.31)	0.2414	792 (280)	1.68 (1.22, 2.32)	**0.0015**	611 (93)
	Inverse-U Pattern	0.80 (0.60, 1.07)	0.1279	179 (56)	1.14 (0.70, 1.88)	0.5958	150 (21)
	High-Decreasing	1.20 (0.99, 1.46)	0.0679	366 (162)	1.32 (0.90, 1.94)	0.1524	293 (45)

Cox proportional hazards models were used for all analyses to examine the association between alcohol consumption trajectory groups and time to all-cause mortality. Covariates included age, education level, BMI, current smoking status, SBP, hypertension treatment, and diabetes at the baseline of Phase 2.

[1] HR, hazard ratio; 95% CI, 95% confidence interval.

[2]The alcohol consumption groups were described in Figure 3. The Moderate-Decreasing drinking group included moderate drinkers (<14 g/day for women and <28 g/day for men) who slightly decreased their consumption. The Inverse-U Pattern drinking group comprised participants with varying alcohol intake patterns, while the High-Decreasing drinking group included participants with consistently high intake levels (>28 g/day for women and >40 g/day for men), also showing a decreasing trend.

SUPPLEMENTAL FIGURES

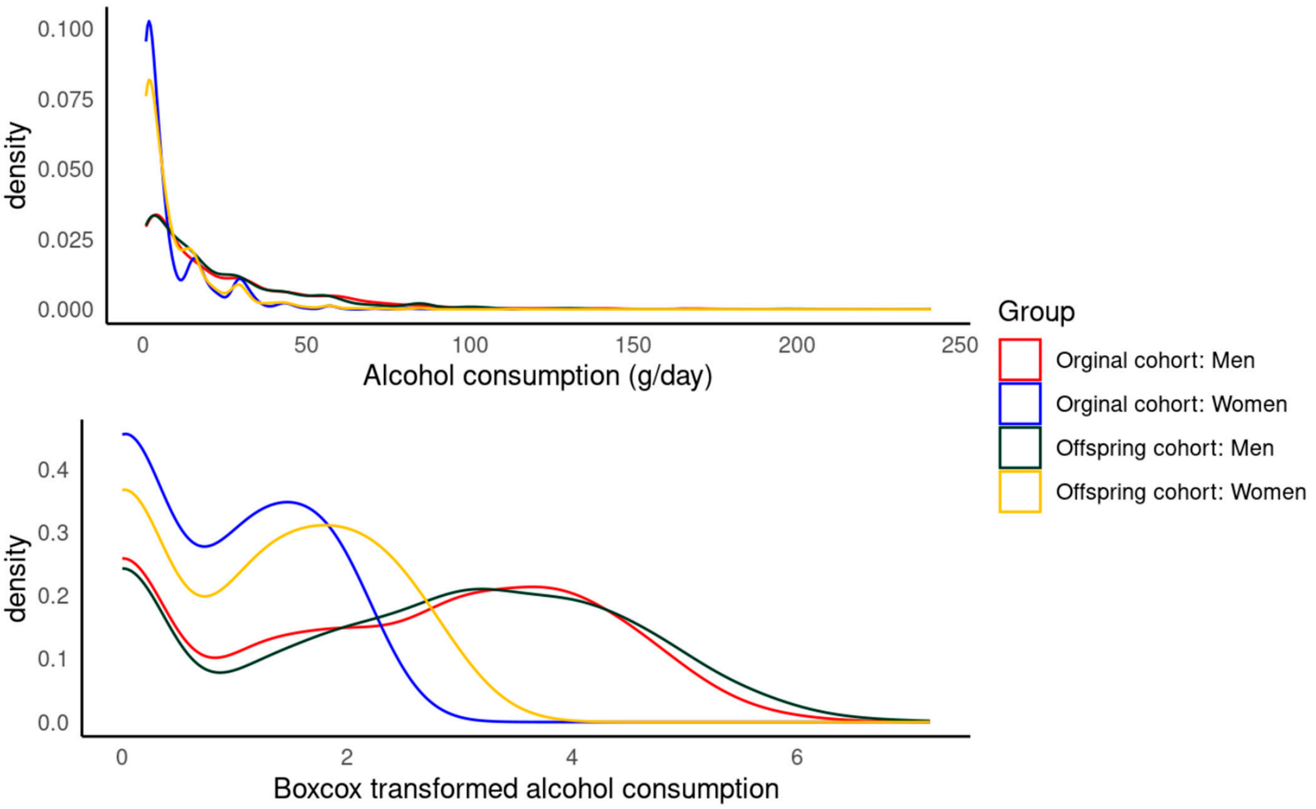

Supplemental Figure S1: Distribution of alcohol consumption. The upper plot shows the distribution of alcohol consumption before transformation, and the lower plot displays the distribution of alcohol consumption after the Box-Cox transformation in the Original and Offspring cohorts of the Framingham Heart Study.

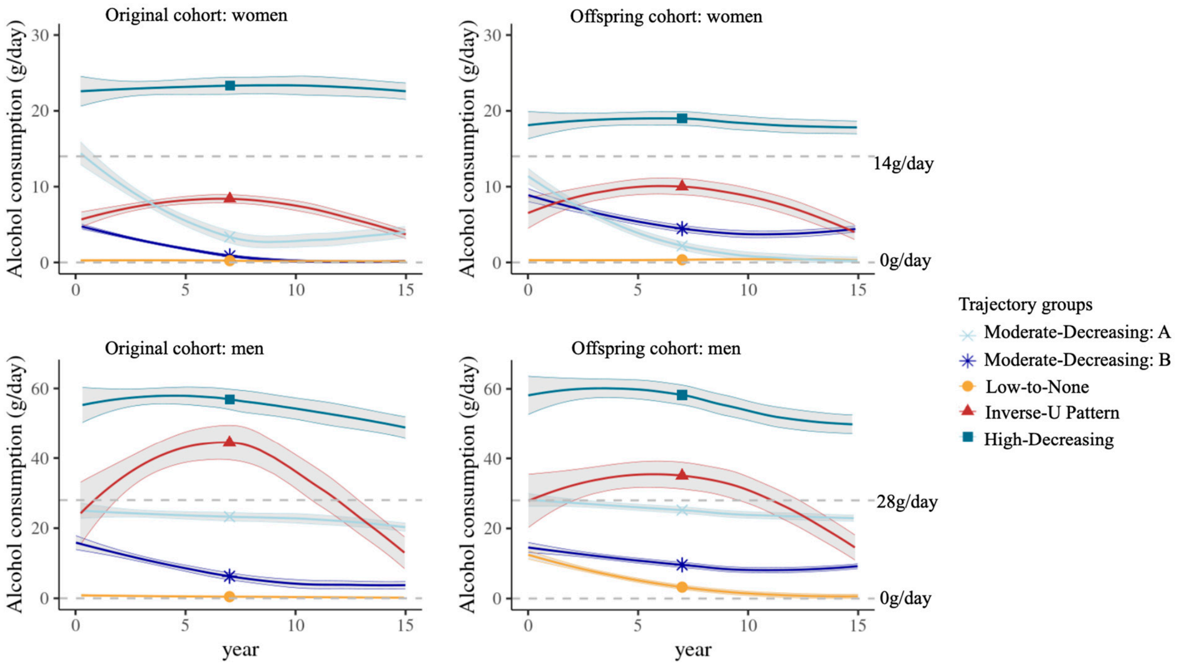

Trajectory groups	Women		Men	
	Original	Offspring	Original	Offspring
Moderate-Decreasing: A	275 (17%)	272 (14%)	372 (33%)	405 (22%)
Moderate-Decreasing: B	208 (13%)	424 (22%)	304 (27%)	453 (25%)
Low-to-None	516 (31%)	476 (25%)	206 (18%)	620 (34%)
Inverse-U Pattern	365 (22%)	241 (12%)	69 (6%)	130 (7%)
High-Decreasing	303 (18%)	555 (28%)	172 (15%)	204 (11%)

Supplemental Figure S1. Five trajectory groups of alcohol consumption in sex- and cohort-specific analysis. A growth mixture model was applied to identify cohort-specific and sex-specific alcohol consumption trajectories. The number and percentage of participants for each trajectory group are presented in the table below.

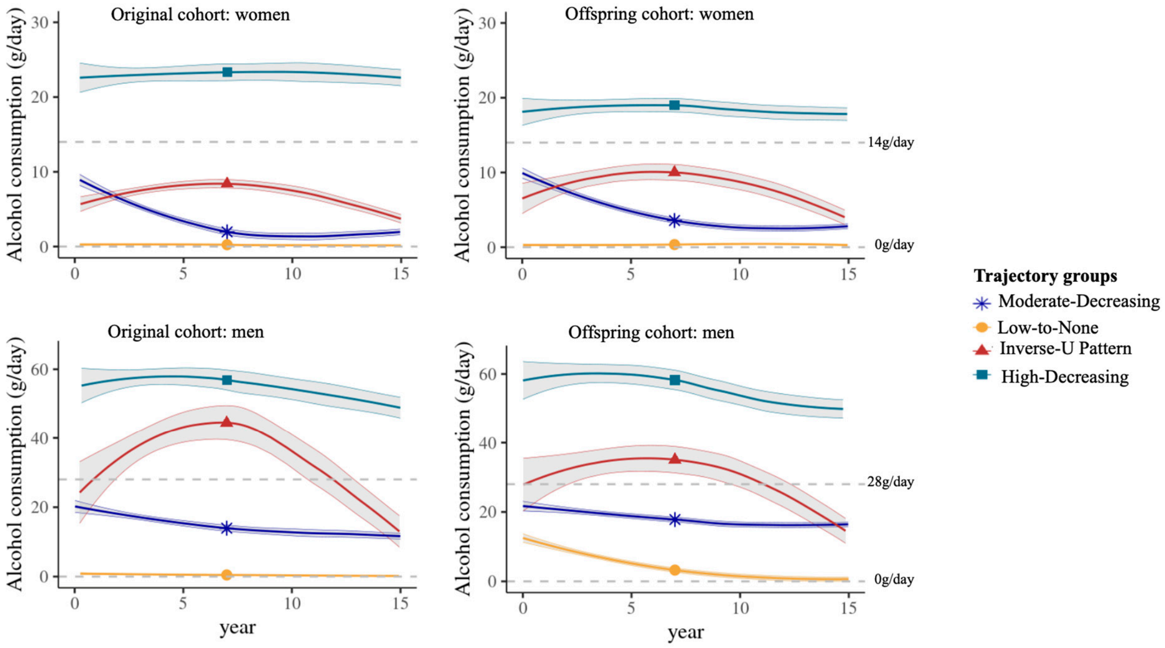

Trajectory groups	Women		Men	
	Original	Offspring	Original	Offspring
Moderate-Decreasing	483 (29%)	696 (35%)	676 (60%)	858 (48%)
Low-to-None	516 (31%)	476 (25%)	206 (18%)	620 (34%)
Inverse-U Pattern	365 (22%)	241 (12%)	69 (6%)	130 (7%)
High-Decreasing	303 (18%)	555 (28%)	172 (15%)	204 (11%)

Supplemental Figure S3. Four trajectory groups of alcohol consumption in sex- and cohort-specific analysis. We merged two trajectory groups, the Moderate-Decreasing A and Moderate-Decreasing B groups (see Supplemental Figure 2), into Moderate-Decreasing drinking group, results in 4 trajectory groups of alcohol consumption in sex- and cohort-specific analysis. A growth mixture model was applied to identify cohort-specific and sex-specific alcohol consumption trajectories. The number and percentage of participants for each trajectory group are presented in the table below.

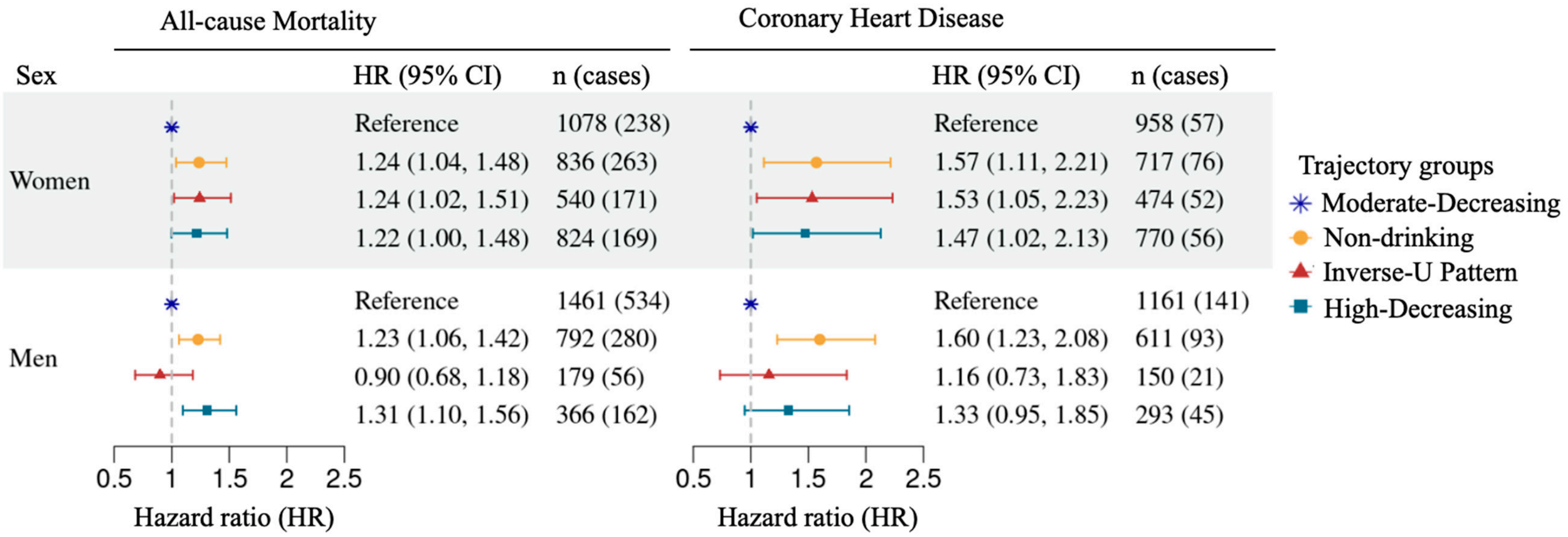

Supplemental Figure S2. Unadjusted sex-stratified association analysis between alcohol consumption trajectory groups with all-cause mortality and incident CHD. Cox proportional hazards regression models were used to quantify associations over a 10-year follow-up period. The alcohol consumption groups were described in Figure 3. Covariates were not adjusted in association analyses. The Moderate-Decreasing drinking group included moderate drinkers (<14 g/day for women and <28 g/day for men) who slightly decreased their consumption. The Inverse-U Pattern drinking group comprised participants with varying alcohol intake patterns, while the High-Decreasing drinking group included participants with consistently high intake levels (>28 g/day for women and >40 g/day for men), also showing a decreasing trend. HR, hazard ratio. 95% CI, 95% confidence interval. n (cases), the total number of participants in a trajectory group (the number of events in this group).

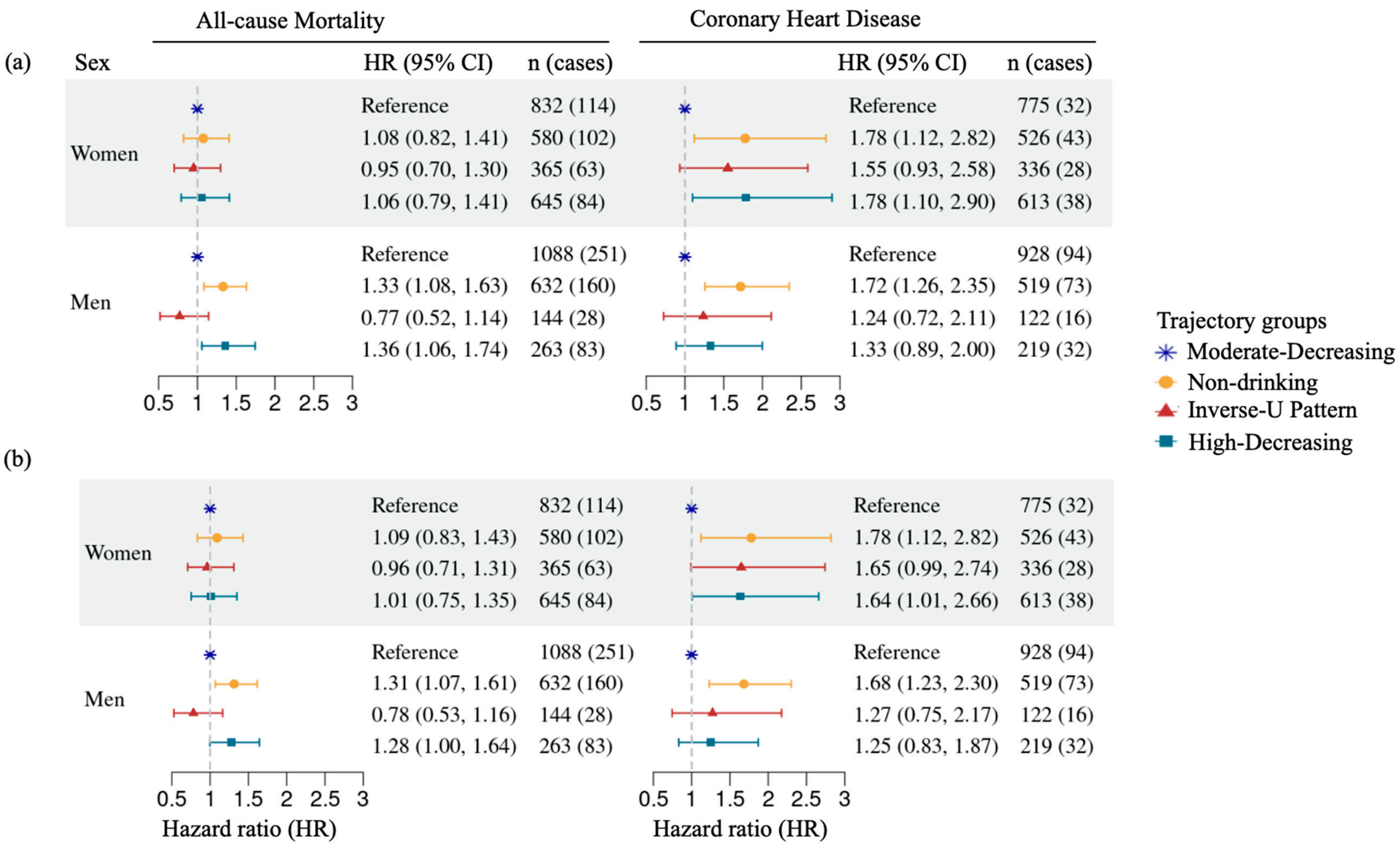

Supplementary Figure S5. **Sex-stratified association analysis of alcohol consumption trajectory groups with all-cause mortality and incident CHD, comparing models with individual covariates versus those adjusted using the ASRS.** Cox proportional hazards regression models were used to quantify associations over a 10-year follow-up period. The same sample size was used, limited to participants with available Atherosclerotic Cardiovascular Disease Risk Score (ASRS) data and all individual covariate

data. Cox proportional hazards regression models were used for all analyses to quantify associations: (a) adjusting for individual covariates, including age, education level, BMI, current smoking status, SBP, hypertension treatment, and diabetes at the baseline of Phase 2.
(b) adjusting for ASRS as the covariate instead of individual covariates. The alcohol consumption groups were described in Figure 3. The Moderate-Decreasing drinking group included moderate drinkers (<14 g/day for women and <28 g/day for men) who slightly decreased their consumption. The Inverse-U Pattern drinking group comprised participants with varying alcohol intake patterns, while the High-Decreasing drinking group included participants with consistently high intake levels (>28 g/day for women and >40 g/day for men), also showing a decreasing trend. HR, hazard ratio. 95% CI, 95% confidence interval. n (cases), the total number of participants in a trajectory group (the number of events in this group).

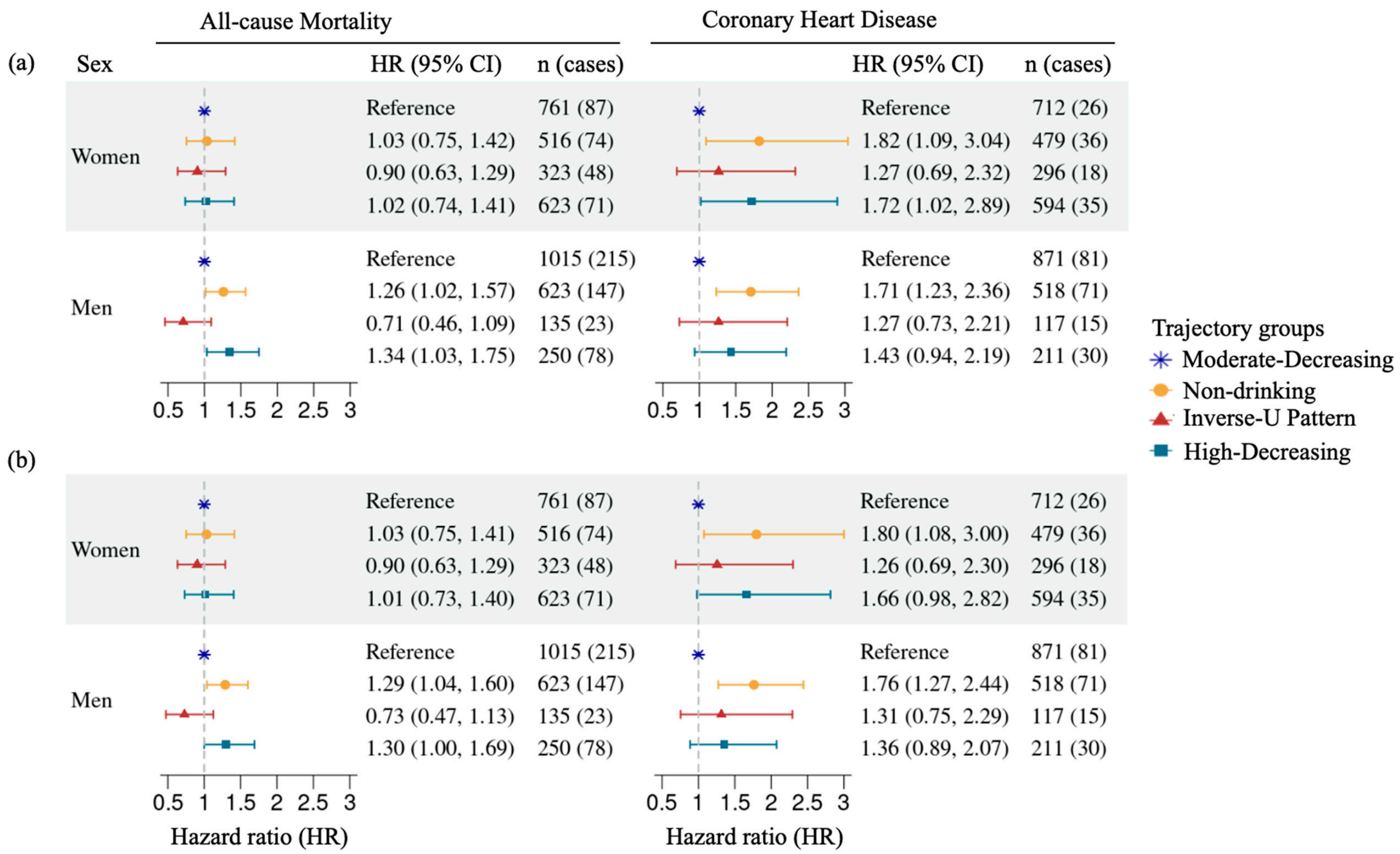

Supplementary Figure S6. **Sex-stratified association analyses of alcohol consumption trajectory groups with all-cause mortality and incident CHD, comparing models with individual covariates versus those adjusted the FRS.** Cox proportional hazards regression models were used to quantify associations over a 10-year follow-up period. The same sample size was used,

limited to participants with available the Framingham Risk Score (FRS) data and all individual covariate data. Cox proportional hazards regression models were used for all analyses to quantify the associations: (a) adjusting for individual covariates, including age, education level, BMI, current smoking status, SBP, hypertension treatment, and diabetes at the baseline of Phase 2; (b) adjusting for FRS instead of individual covariates. The alcohol consumption groups were described in Figure 3. The Moderate-Decreasing drinking group included moderate drinkers (<14 g/day for women and <28 g/day for men) who slightly decreased their consumption. The Inverse-U Pattern drinking group comprised participants with varying alcohol intake patterns, while the High-Decreasing drinking group included participants with consistently high intake levels (>28 g/day for women and >40 g/day for men), also showing a decreasing trend. HR, hazard ratio. 95% CI, 95% confidence interval. n (cases), the total number of participants in a trajectory group (the number of events in this group).

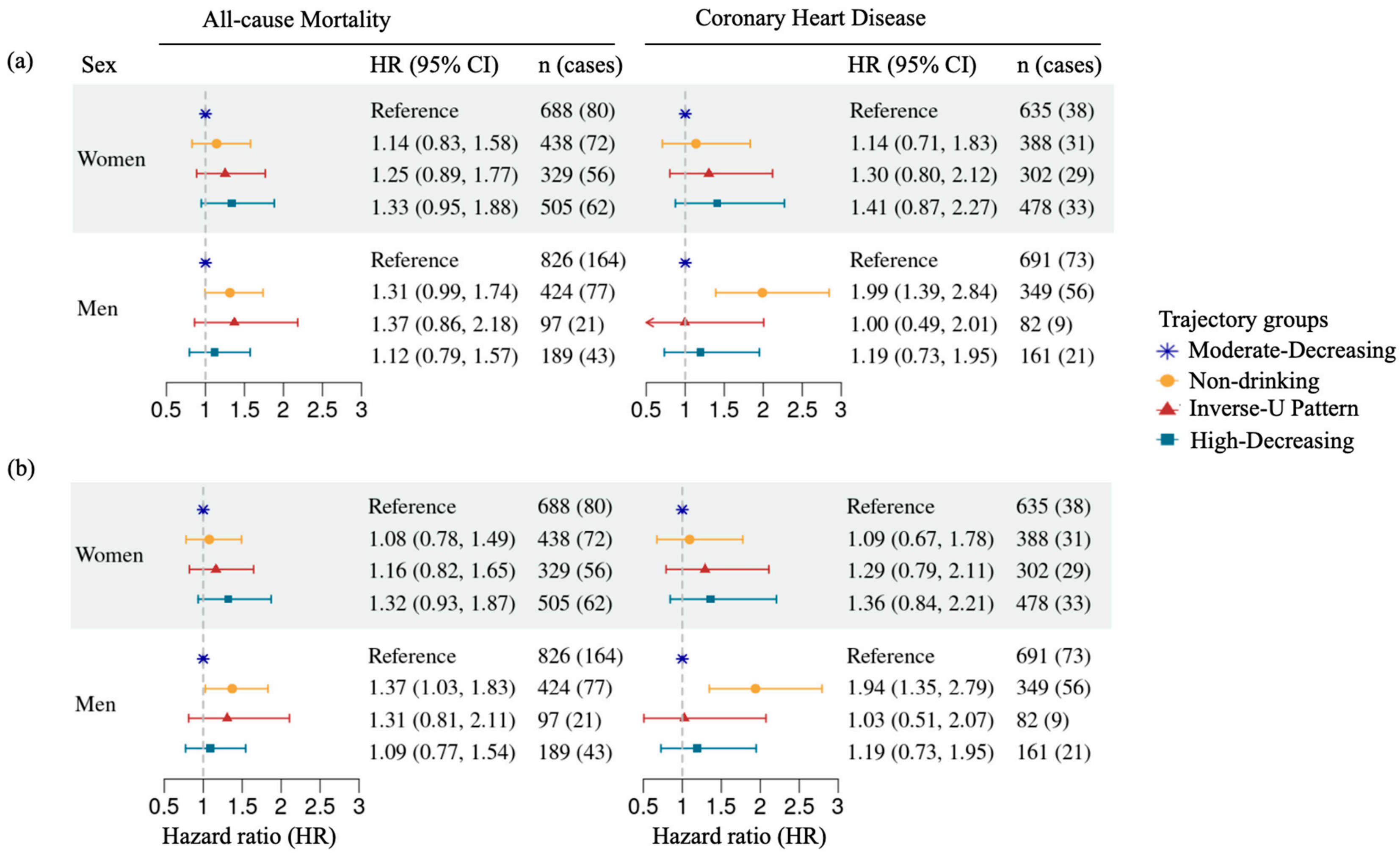

(a)
All-cause Mortality
Coronary Heart Disease
Sex
HR (95% CI)
n (cases)
Women
Reference 688 (80)
1.14 (0.83, 1.58) 438 (72)
1.25 (0.89, 1.77) 329 (56)
1.33 (0.95, 1.88) 505 (62)
Reference 635 (38)
1.14 (0.71, 1.83) 388 (31)
1.30 (0.80, 2.12) 302 (29)
1.41 (0.87, 2.27) 478 (33)
Men
Reference 826 (164)
1.31 (0.99, 1.74) 424 (77)
1.37 (0.86, 2.18) 97 (21)
1.12 (0.79, 1.57) 189 (43)
Reference 691 (73)
1.99 (1.39, 2.84) 349 (56)
1.00 (0.49, 2.01) 82 (9)
1.19 (0.73, 1.95) 161 (21)
0.5 1 1.5 2 2.5 3
(b)
Women
Reference 688 (80)
1.08 (0.78, 1.49) 438 (72)
1.16 (0.82, 1.65) 329 (56)
1.32 (0.93, 1.87) 505 (62)
Reference 635 (38)
1.09 (0.67, 1.78) 388 (31)
1.29 (0.79, 2.11) 302 (29)
1.36 (0.84, 2.21) 478 (33)
Men
Reference 826 (164)
1.37 (1.03, 1.83) 424 (77)
1.31 (0.81, 2.11) 97 (21)
1.09 (0.77, 1.54) 189 (43)
Reference 691 (73)
1.94 (1.35, 2.79) 349 (56)
1.03 (0.51, 2.07) 82 (9)
1.19 (0.73, 1.95) 161 (21)
0.5 1 1.5 2 2.5 3
Hazard ratio (HR)
Trajectory groups
Moderate-Decreasing
Non-drinking
Inverse-U Pattern
High-Decreasing

Supplementary Figure S7. Sex-stratified association analyses of alcohol consumption trajectory groups with all-cause mortality and incident CHD, comparing the models adjusted for primary individual covariates with those adjusted for additional covariates. Cox proportional hazards regression models were used to quantify associations over a 10-year follow-up period. In models adjusted for primary covariates, the covariates included age, education level, BMI, current smoking status, SBP, hypertension treatment, and diabetes at the baseline of Phase 2. Additional covariates included physical activity records, marital status, occupation category, and cancer records. (a) Models adjusted for primary covariates. (b) Models adjusted for additional covariates plus the primary covariates. The same sample size was used, limited to participants with both the primary and additional covariates. The alcohol consumption groups were described in Figure 3. The Moderate-Decreasing drinking group included moderate drinkers (<14 g/day for women and <28 g/day for men) who slightly decreased their consumption. The Inverse-U Pattern drinking group comprised participants with varying alcohol intake patterns, while the High-Decreasing drinking group included participants with consistently high intake levels (>28 g/day for women and >40 g/day for men), also showing a decreasing trend. HR, hazard ratio. 95% CI, 95% confidence interval. n (cases), the total number of participants in a trajectory group (the number of events in this group).

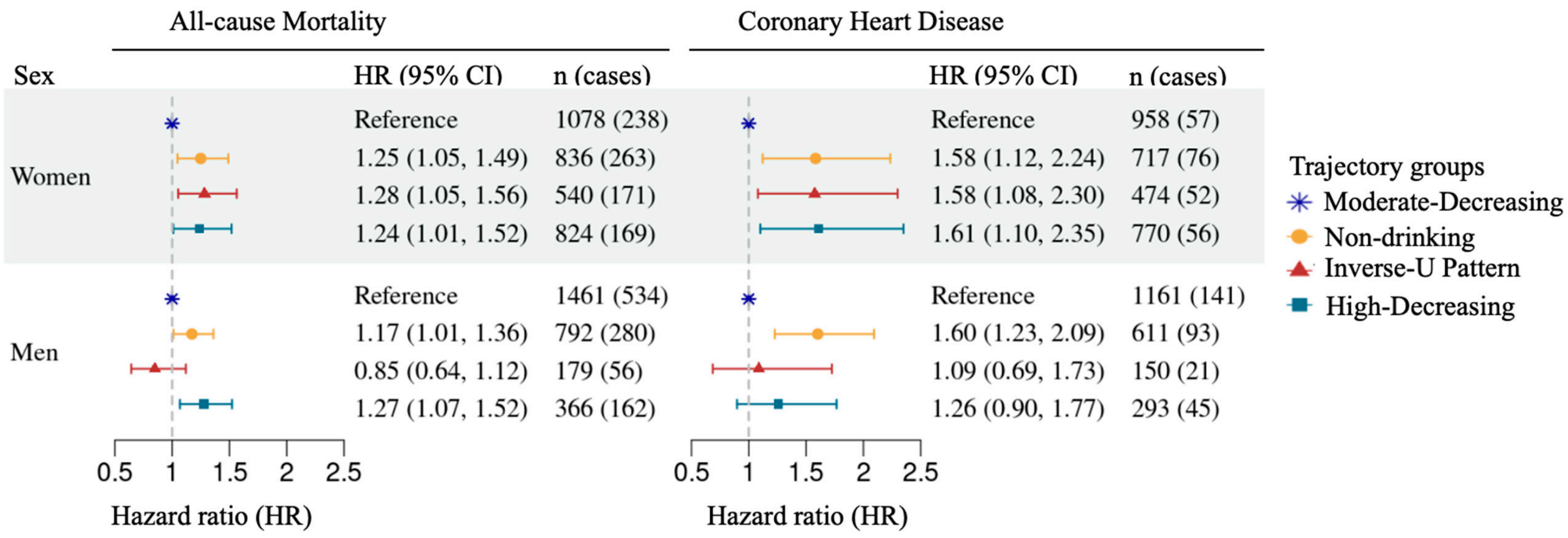

Supplementary Figure S8. **Sex-stratified association analyses of alcohol consumption trajectory groups with all-cause mortality and incident CHD, adjusting for family ID.** The results without adjusting for family ID was displayed in Figure 4 in the main text. Cox proportional hazards regression models were used to quantify associations over a 10-year follow-up period. Covariates included age, education level, BMI, current smoking status, SBP, hypertension treatment, and diabetes at the baseline of Phase 2. The alcohol consumption groups were described in Figure 3. The Moderate-Decreasing drinking group included moderate drinkers (<14 g/day for women and <28 g/day for men) who slightly decreased their consumption. The Inverse-U Pattern drinking group comprised participants with varying alcohol intake patterns, while the High-Decreasing drinking group included participants with consistently high intake levels (>28 g/day for women and >40 g/day for men), also showing a decreasing trend. HR, hazard ratio. 95% CI, 95% confidence interval. n (cases), the total number of participants in a trajectory group (the number of events in this group).

REFERENCES

1. Tan, Z.S., et al., *Physical Activity, Brain Volume, and Dementia Risk: The Framingham Study.* J Gerontol A Biol Sci Med Sci, 2017. **72**(6): p. 789-795.
2. Wang, X., et al., *Association of Depressive Symptom Trajectory With Physical Activity Collected by mHealth Devices in the Electronic Framingham Heart Study: Cohort Study.* JMIR Ment Health, 2023. **10**: p. e44529.
